# Bach1 promotes muscle regeneration through repressing Smad-mediated inhibition of myoblast differentiation

**DOI:** 10.1371/journal.pone.0236781

**Published:** 2020-08-10

**Authors:** Katsushi Suzuki, Mitsuyo Matsumoto, Yasutake Katoh, Liang Liu, Kyoko Ochiai, Yuta Aizawa, Ryoichi Nagatomi, Hiroshi Okuno, Eiji Itoi, Kazuhiko Igarashi

**Affiliations:** 1 Department of Biochemistry, Tohoku University Graduate School of Medicine, Sendai, Miyagi, Japan; 2 Department of Orthopaedic Surgery, Tohoku University Graduate School of Medicine, Sendai, Miyagi, Japan; 3 Center for Regulatory Epigenome and Diseases, Tohoku University Graduate School of Medicine, Sendai, Miyagi, Japan; 4 Japan Agency for Medical Research and Development, Chiyoda, Tokyo, Japan; 5 Department of Medicine and Science in Sports and Exercise, Tohoku University Graduate School of Medicine, Sendai, Miyagi, Japan; 6 Department of Orthopaedic Surgery, Tohoku Rosai Hospital, Sendai, Miyagi, Japan; University of Minnesota Medical School, UNITED STATES

## Abstract

It has been reported that *Bach1*-deficient mice show reduced tissue injuries in diverse disease models due to increased expression of heme oxygenase-1 (HO-1)that possesses an antioxidant function. In contrast, we found that *Bach1* deficiency in mice exacerbated skeletal muscle injury induced by cardiotoxin. Inhibition of Bach1 expression in C2C12 myoblast cells using RNA interference resulted in reduced proliferation, myotube formation, and myogenin expression compared with control cells. While the expression of HO-1 was increased by Bach1 silencing in C2C12 cells, the reduced myotube formation was not rescued by HO-1 inhibition. Up-regulations of Smad2, Smad3 and FoxO1, known inhibitors of muscle cell differentiation, were observed in *Bach1*-deficient mice and *Bach1*-silenced C2C12 cells. Therefore, Bach1 may promote regeneration of muscle by increasing proliferation and differentiation of myoblasts.

## Introduction

Skeletal muscle injury is a common disorder, especially among athletes [1–7]. After the injury, sports activities must be restricted from athletes to allow collagen integration and to prevent complications including reinjury even though they demand to make a comeback as soon as possible [8]. Therefore, it is critical to understand the regeneration process of skeletal muscle to solve injury-related problems.

Recent studies have revealed a negative function of BTB and CNC homology 1 (Bach1)to exacerbate tissue damages in multiple disease models [9–15]. For example, *Bach1*-deficient mice show less area of infarction after an ischemia-reperfusion model of the heart [9]. Bach1 is a transcription regulatory protein that is broadly expressed in diverse range of tissues in both mice and human [16, 17]. Bach1 forms heterodimers with small Maf proteins, and represses the expression of target genes by binding a subset of Maf recognition elements (MAREs) in the promoter or enhancer regions of these genes [18, 19]. Among the target genes of Bach1 is *Hmox1* encoding heme oxygenase-1 (HO-1), which is important for the protection against oxidative stress [20]. De-repression of *Hmox1* in *Bach1*-deficient mice has been suggested to contribute to reductions of tissue injuries in diverse disease models [9–15]. Bach1 also represses a portion of p53 target genes by interacting with p53 and histone deacetylase-1 (HDAC1), resulting in inhibition of p53-mediated cellular senescence [21]. Mapping of Bach1 binding sites on the mouse genome has revealed that Bach1 represses adipocyte differentiation activity of fibroblasts as well [22]. However, little has been known on the function of Bach1 in the skeletal muscle system.

Skeletal muscle injury is aggravated by oxidative stress [23, 24]. Cellular senescence of satellite cell, the major contributor for muscle repair, precludes efficient muscle regeneration [25, 26]. Therefore, we formulated several hypotheses that Bach1 would inhibit muscle regeneration. Bach1 may increase oxidative stress by repressing the expression of *Hmox1*. Alternatively, Bach1 would promote muscle regeneration through reducing senescence of satellite cells. There is also the possibility that new Bach1 target genes would modulate muscle repair. To investigate these possibilities, we examined the putative function of Bach1 in muscle regeneration after cardiotoxin-induced injury byusing *Bach1*-deficient mice. To identify underlying mechanisms, we performed knockdown of *Bach1*using the myoblast cell line C2C12. With these approaches, we revealed animportant role of Bach1 for promoting muscle regeneration.

## Results

### The skeletal muscle of *Bach1*-deficient mice presents normal structure and histology

**Fig 1:**
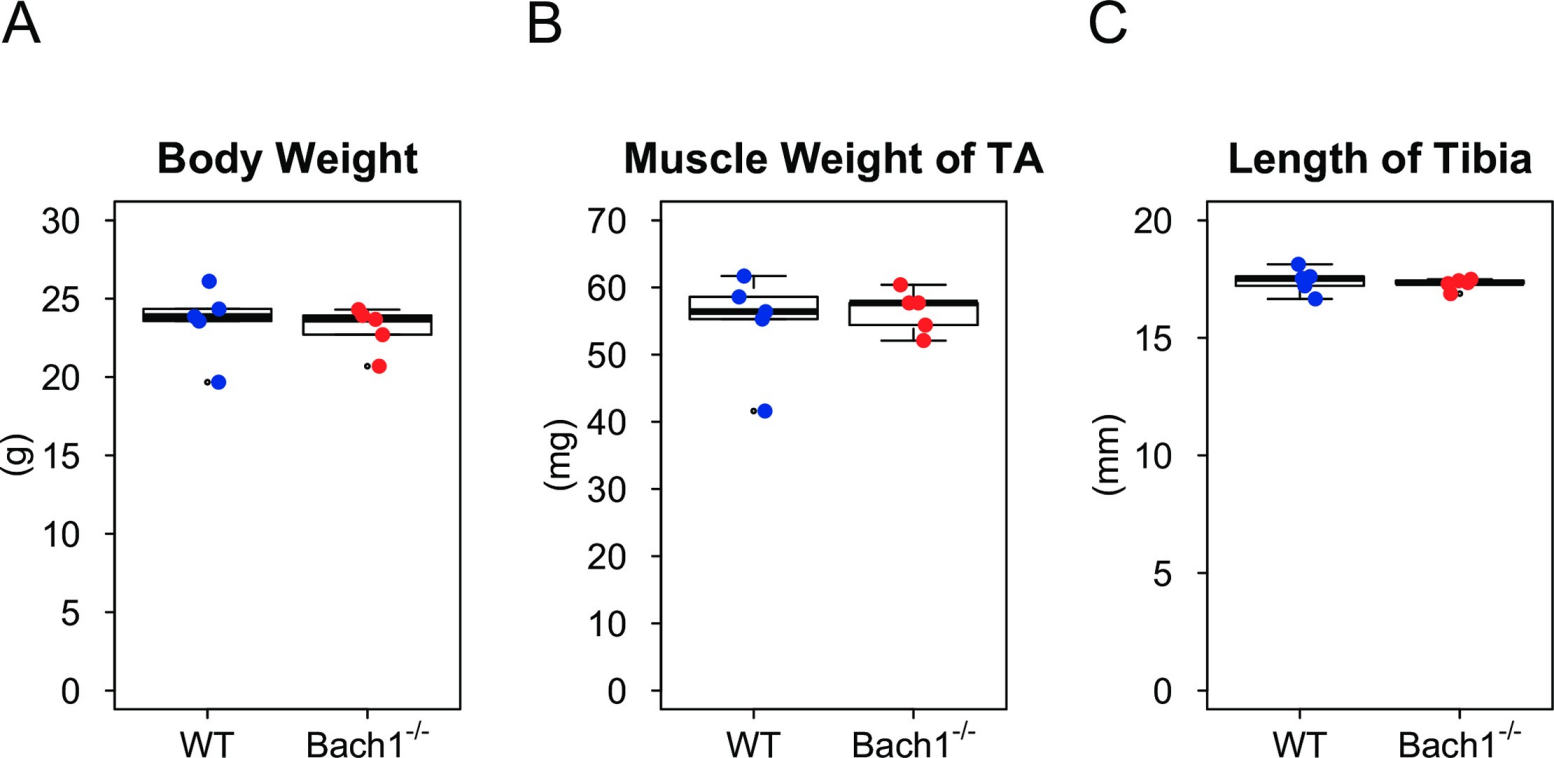
Bach1 is dispensable for muscle growth. (A-C) Box plots showing body weight (A), muscle weight (B) and length of tibia (C) of uninjured Bach1-deficient and WT mice.(*n* = 5).

**Fig 2:**
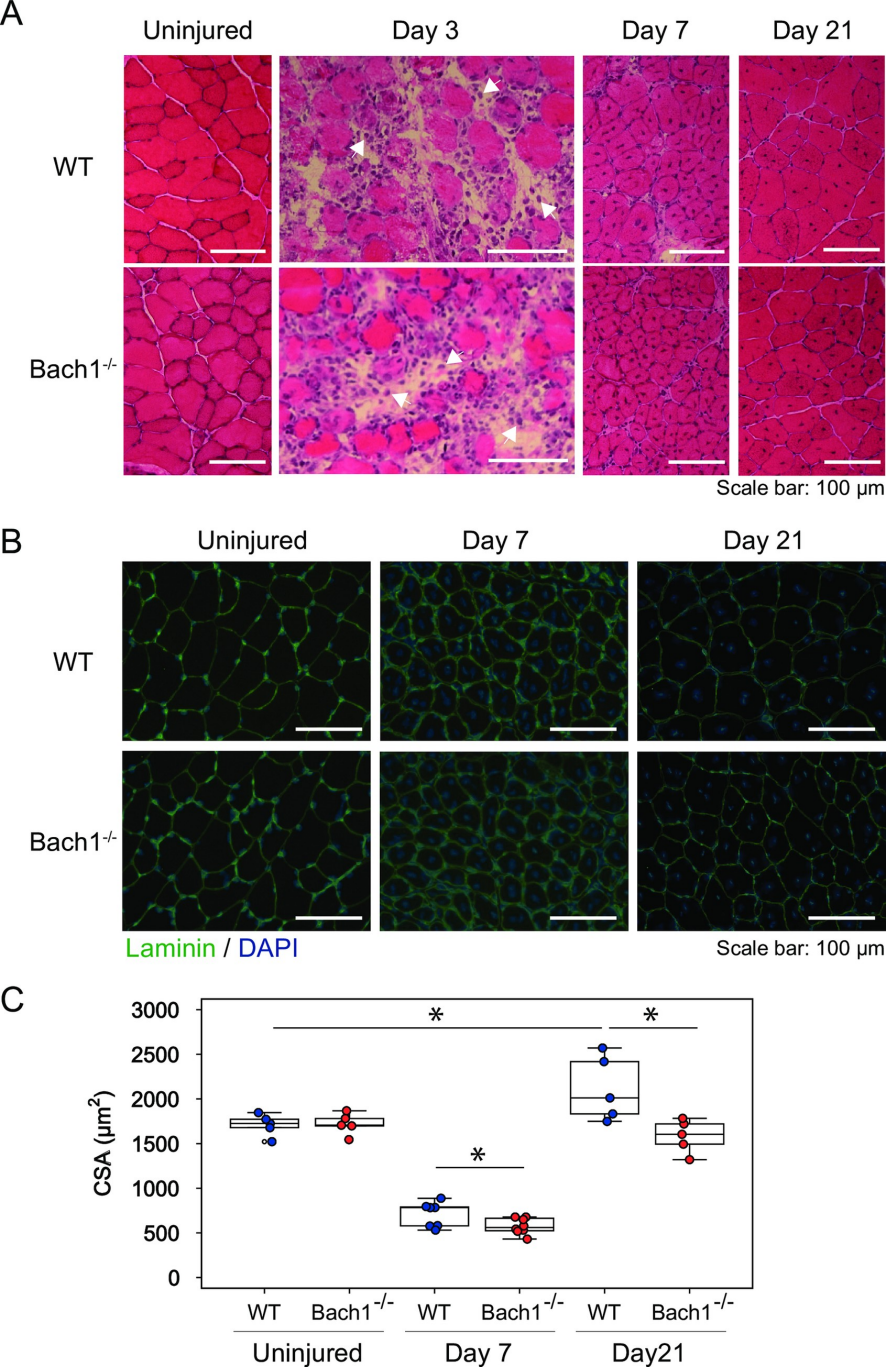
Muscle regeneration is impaired in *Bach1*-deficient mice. (A) HE staining of tibialis anterior muscle sections from *Bach1*-deficient and WT mice 3, 7, or 21 days after muscle injury. Uninjured samples are also shown. Mononuclear cells infiltrated on day 3 (arrow heads). (B) Immunofluorescence staining with anti-Laminin antibody (green) of tibialis anterior muscle sections of *Bach1*-deficient and WT. Nuclei were stained with DAPI (blue). (C) CSA of injured muscle was calculated using myofibers with central nuclei. (*n* = 5–8; * *p*< 0.05).

As reported before [27], body weight, somatotype, lifespan, and fecundity of *Bach1*-deficient mice were similar to those of wild-type (WT)*m*ice. We evaluated the effect of *Bach1* deficiency on somatotype and skeletal muscle in uninjured mice. No difference was found among the two groups in body weight, length of tibialis anterior, or weight of tibialis anterior (Fig 1A–1C). HE staining of the muscle revealed normal structures in *Bach1*-deficient mice (Fig 2A, left). Using Laminin staining of muscle, we determined cross sectional area (CSA) which indicates myofiber size. The measured CSA oftibialis anterior muscle of *Bach1*-deficient mice was similar to that of WT mice (Fig 2B and 2C). These observations indicate that *Bach1* deficiency does not affect somatotype and skeletal muscle in uninjured mice.

### Bach1 deficiency inhibits skeletal muscle regeneration

To evaluate the role of Bach1 in skeletal muscle injury, we employed a muscle injury model. We injected cardio toxin, snake venom of Najapallida, into tibialis anterior muscle of both *Bach1*-deficient and WT mice [28, 29]. These mice were sacrificed at 3, 7, and 21 days after injury. At 3 days after injury, most of the muscle fibers became deformed and the numbers of intact muscle fibers decreased (Fig 2A). In addition, many mononuclear cells containing little cytoplasm were seen in the stroma. These changes appeared to reflect the degradation phase in a muscle injury [30]. At 7 days after injury, muscle fibers with centrally located nuclei appeared (Fig 2A and 2B). Normal muscle fibers have peripherally located nuclei, but newly regenerated fibers possesscentrally located nuclei, which eventually move to a border of muscle fibers at the end of regeneration period [31]. We measured CSA of injured muscles using Laminin staining (Fig 2B and 2C). Only muscle fibers having centrally located nuclei were chosen for measurement to compare regeneration activities in these mice. At 7 days, CSA values decreased less than half compared with non-injured muscle (Fig 2C). The mean CSA value of *Bach1*-deficient mice was significantly lower than that of WT miceat 7 days (Fig 2C). At 21 day, CSA was substantially improved in both types of mice but remained lower in *Bach1*-deficient mice than WT mice, and the difference of CSA became larger (Fig 2C). These results show that muscle regeneration ability was impaired in *Bach1*-deficient mice.

### Bach1 protein is up-regulated at early stage of muscle injury

**Fig 3:**
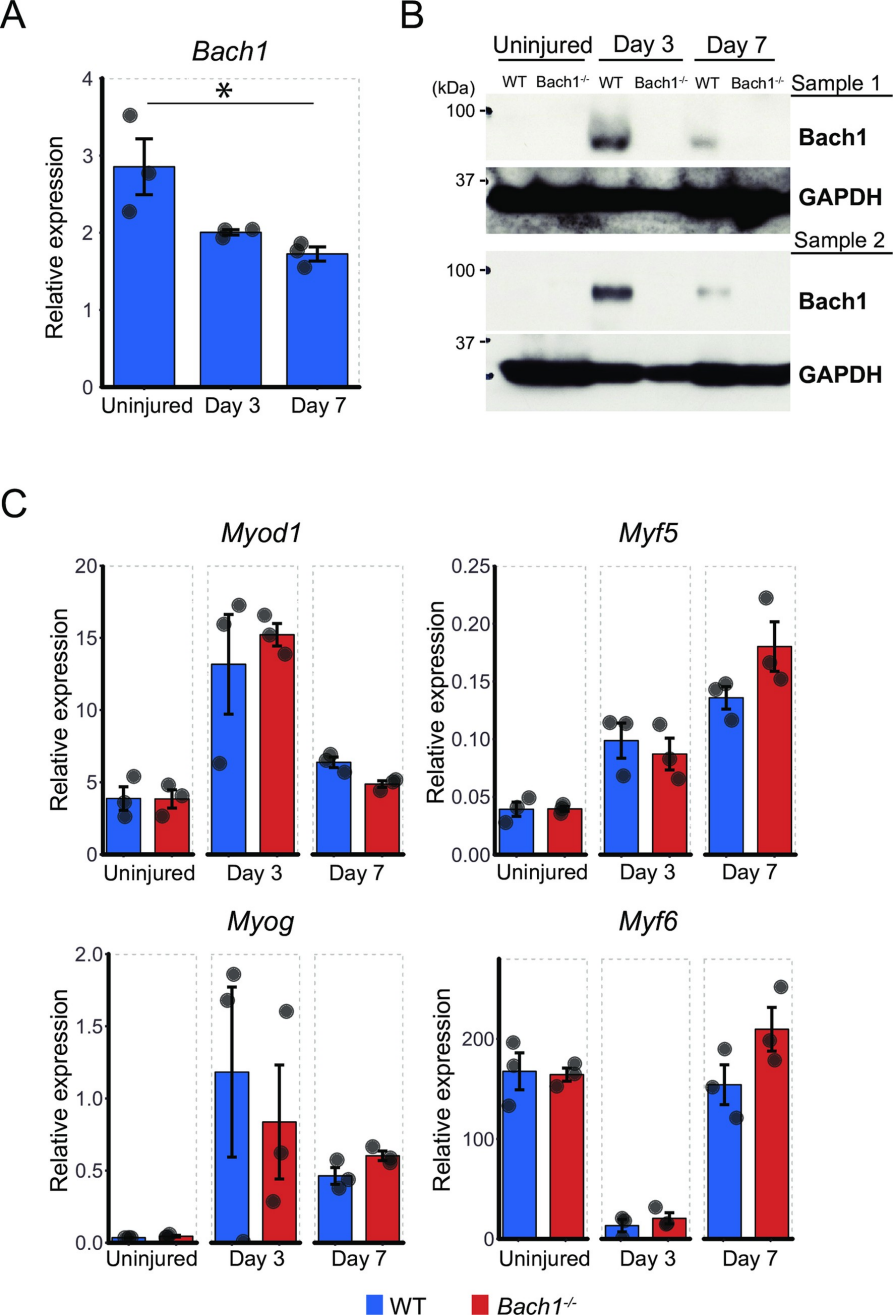
Bach1 protein increases after skeletal muscle injury. (A) Expression level of Bach1 mRNA measured by RT-qPCR in muscle of WT mice at indicated time points after muscle injury (*n* = 3; * *p*< 0.05). (B) Western blotting of muscle of *Bach1*-deficient and WT mice as above with antibodies against Bach1, or GAPDH as an internal control. (C) Expression of *Myod1* (MyoD), *Myf5*, *Myog* (Myogenin) and *Myf6* (MRF4) mRNA measured by RT-qPCR in muscle of *Bach1*-deficient and WT mice as above. (*n* = 3).

Since muscle regeneration was aggravated in *Bach1*-deficient mice, we evaluated mRNA and protein expression levels of Bach1 in the skeletal muscle of WT mice after muscle injury. Muscle of *Bach1*-deficient mice was used as a negative control. The quantity of Bach1 mRNA was gradually decreased after muscle injury (Fig 3A). The amount of Bach1 protein was drastically increased on 3days after muscle injury, and decreased again along the progression of muscle regeneration (Fig 3B). These results were consistent with the above interpretation that Bach1played a regulatory role in the early phase of muscle regeneration.

To obtain a clue to understand the molecular alterations in the reduced regeneration of Bach1-deficient muscle, we checked the expression of four genes (*MyoD*, *Myf5*, *Myog*, and *Myf6*) belonging to the muscle regulatory factors (MRFs) using mRNA of *Bach*1-deficient and WT mice. Three of them (*Myod1*, *Myf5* and *Myog*) were induced by the cardiotoxin treatment and there was no significant difference in the expression of these MRFs mRNA in the skeletal muscle between *Bach1*-deficient and WT mice (Fig 3C). Myf6 mRNA showed a transient reduction irrespective of the genotypes of the mice. These results suggested that Bach1 regulated muscle cell regeneration without affecting the expression of MRFs.

**Fig 4:**
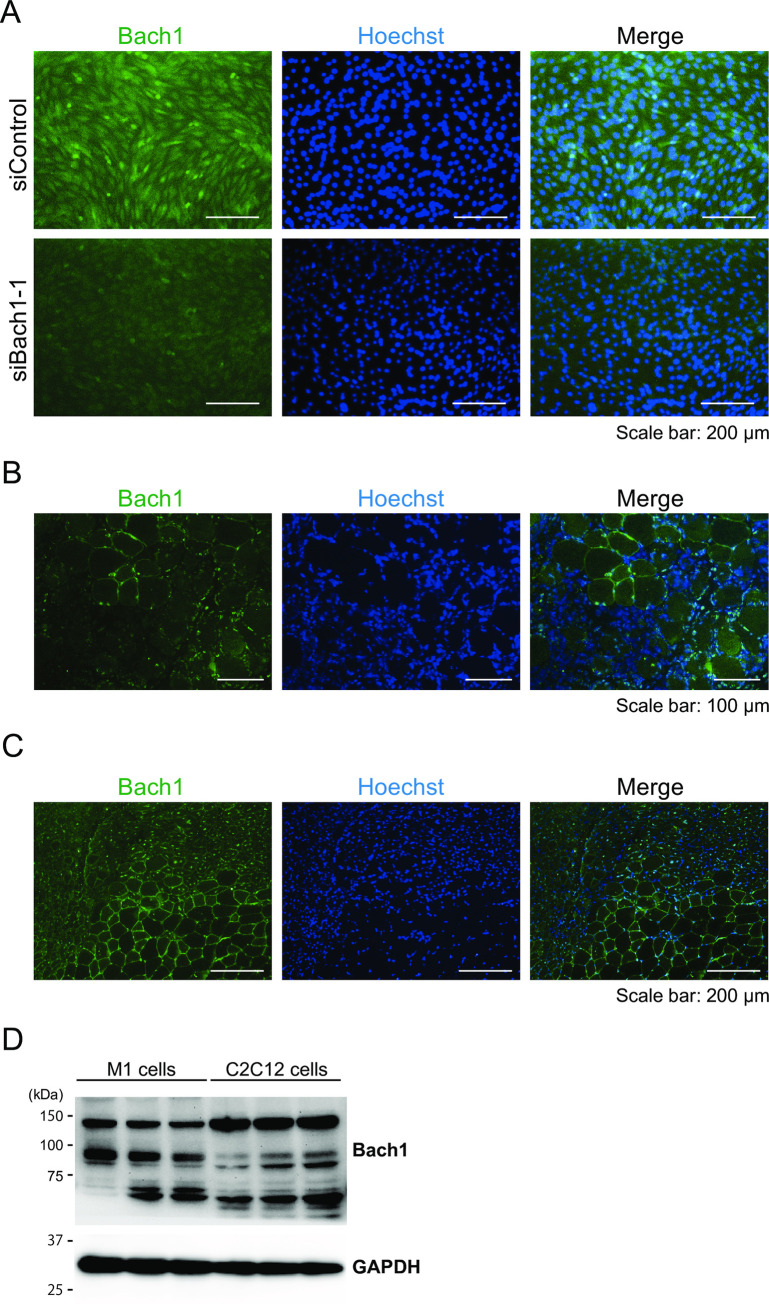
Bach1 protein increases in regenerated muscle fibers. (A) Immunofluorescence staining with anti-Bach1 antibody (green) of C2C12 cells transfected with Bach1 siRNA (siBach1-1) or control siRNA. Nuclei were stained with Hoechst (blue). (*n* = 3). (B) Immunofluorescence staining with anti-Bach1 antibody (green) of tibialis anterior muscle sections from WT mice 3 days after muscle injury. Nuclei were stained with Hoechst (blue) (*n* = 3). (C) Immunofluorescence staining with anti-Bach1 antibody (green) of tibialis anterior muscle sections from WT mice 7 days after muscle injury. Nuclei were stained with Hoechst (blue) (*n* = 6). (D) Western blotting of M1 cells and C2C12 cells 6 days after inducing differentiation.

Next, we tried to determine whether Bach1 protein was expressed in regenerating muscle cells. We first confirmed the specificity of anti-Bach1 antibody for immunostaining (Fig 4A). We then examined the expression of Bach1 protein in injured muscle. Bach1 was upregulated in small mononuclear cells 3 days after the injury (Fig 4B), which appeared to be either myoblast or immune cells. Bach1 protein was clearly detected in regenerating muscle cells with centrally located nuclei7 days after the injury (Fig 4C). We compared Bach1 protein levels between C2C12 cells and myeloid leukemia cell line M1 cells and found that the amount of Bach1 protein was more in M1 cells than in C2C12 cells which were induced toward muscle cell differentiation for 6 days (Fig 4D). However, Bach1 protein was decreased by 5 days after muscle differentiation of C2C12 cells (see below). Taking these results together, we surmised that Bach1 played a role in regenerating muscle cells.

### Bach1 promotes proliferation and differentiation of myoblastic cell line

**Fig 5:**
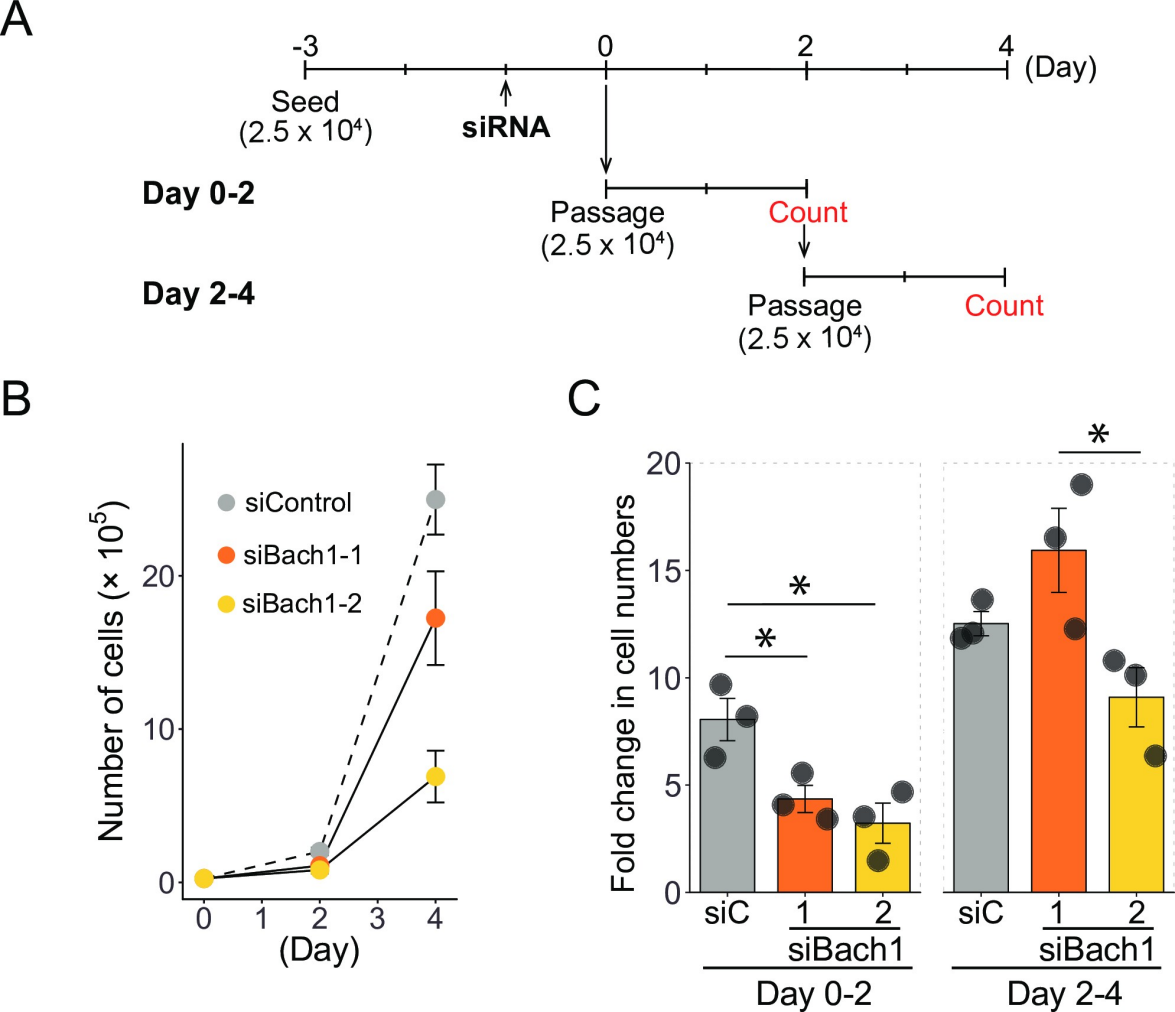
Bach1 is necessary for proliferation of C2C12 cells. (A) Experimental scheme of the proliferation analysis. Cells were cultured in 12-well plate. (B) Cell numbers of C2C12 cells transfected with Bach1 siRNA (siBach1-1 and siBach1-2) or control siRNA. (*n* = 3). (C) Proliferation rates of C2C12 cells with Bach1 silencing or control C2C12 cells at indicated periods. (*n* = 3; * *p*< 0.05).

Regeneration of muscle involves satellite cells and their muscle differentiation. We used C2C12 myoblast cell line as a model of satellite cell differentiation [32] and performed gene silencing experiments to reveal functions of Bach1 in myoblasts and their muscle cell differentiation. C2C12 cells can be induced to differentiate after growing to confluent and changing medium to a lower serum conditions [33]. After transfected with short interfering RNAs targeting Bach1 (siBach1-1 and siBach1-2) or a control RNA (siControl), proliferation of C2C12 cells were monitored (Fig 5A). Upon Bach1 silencing, the cells showed significantly decreased proliferation especially at 2 days after the transfection (Fig 5B and 5C), indicating a critical role of Bach1 in the proliferation of C2C12 cells.

**Fig 6:**
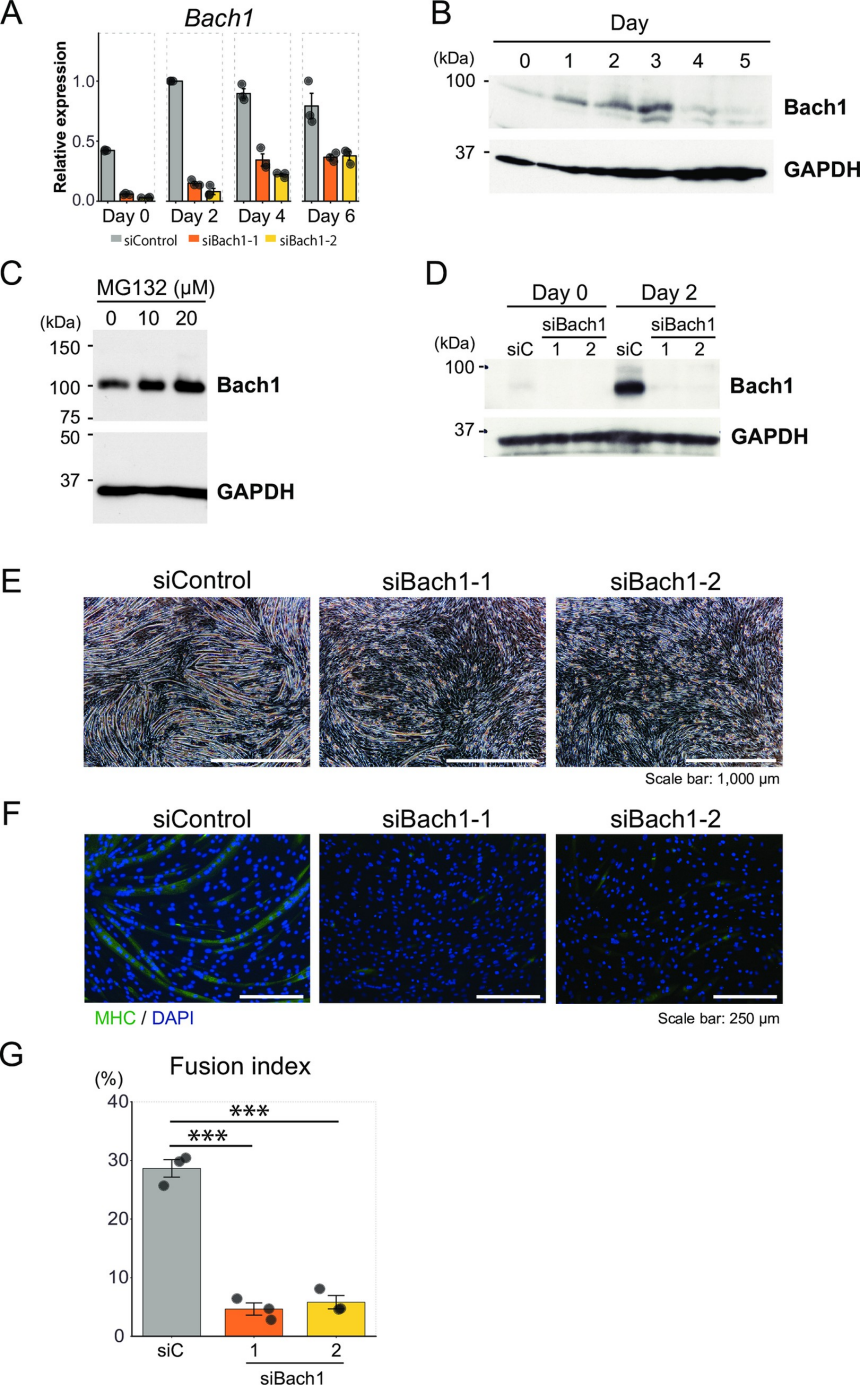
Bach1 is required for myoblasts differentiation of C2C12 cells. (A) Expression levels of *Bach1* mRNA were measured by RT-qPCR in C2C12 cells transfected with indicated siRNA before (day 0) and after inducing differentiation (day 2, 4 and 6). (*n* = 3). (B) Western blotting of C2C12 cells before (day 0) and after inducing differentiation(day 1–5). (C) Western blotting of MG132-treated and control C2C12 cells. (D) Western blotting of Bach1 in indicated C2C12 cells before (day 0) and after inducing differentiation (day 2). (E) Morphology of C2C12 cells 5 days after inducing differentiation. (F) Immunohistochemical staining of MHC and nuclei (DAPI) of Bach1-silenced and control C2C12 cells 6 days after inducing differentiation. (G) Fusion index after 6 days. (*n* = 3; *** *p* < 0.001).

In the early stage of muscle injury, the amount of Bach1 protein increased (see Fig 3B) especially in small mononuclear cells (Fig 4B). To confirm that the upregulated expression of Bach1 protein after muscle injury was derived from not only immune cells but also muscular cells, we determined amounts of Bach1 mRNA and protein after inducing differentiation of C2C12 cells. The amounts of Bach1 mRNA increased in C2C12 cells after differentiation and remained high (Fig 6A). While Bach1 protein was also induced upon differentiation, it was decreased by 4 days after induction of differentiation (Fig 6B). These different kinetics of mRNA and protein led us to hypothesize that amount of Bach1 protein was controlled by proteosomal degradation. We therefore used the proteasome inhibitor MG132 and found that protein level of Bach1 increased in MG132-treated C2C12 cells (Fig 6C). These results indicated that the expression of Bach1 was increased upon muscle differentiation and suggested that the increased expression of Bach1 in injured muscle was derived from myogenic cells at the differentiated stage. These observations do not exclude the possibility that Bach1 was also induced in non-muscle cells in the injured region. Bach1 protein may be regulated upon muscle differentiation by a post-translational mechanism as well including proteasome-mediated degradation.

**Fig 7:**
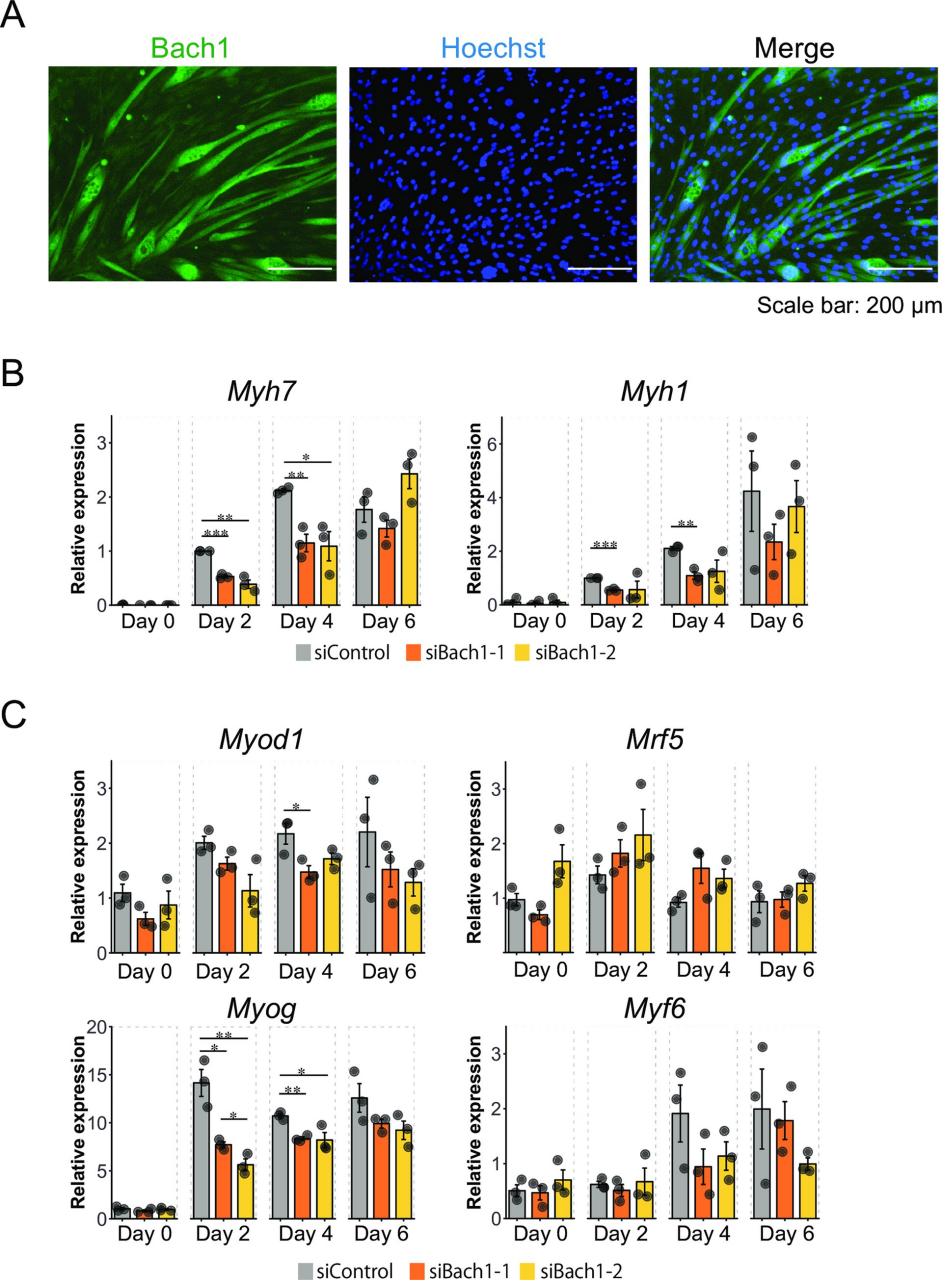
Bach1 regulates *Mhc* and MRFs mRNA expression. (A) Immunohistochemical staining of Bach1 and nuclei (Hoechst) of C2C12 cells 6 days after inducing differentiation. (B, C) Expression levels of *Myh7* (MHC Ⅰ/b) and *Myh1* (MHC Ⅱd/x)mRNA (B) and Myogenic regulatory factors (MRFs: *Myod1* (MyoD), *Myf5*, *Myog* (myogenin) and *Myf6* (Mrf4))mRNA (C) measured by RT-qPCR of Bach1-silenced and control C2C12 cells before (day 0) and after inducing differentiation (day 2, 4 and 6). (*n* = 3; * *p*< 0.05, ** *p*< 0.01, *** *p* < 0.001).

We next examined effects of Bach1 silencing on the differentiation of C2C12 cells (Fig 6D). In contrast to the massive formation of myotubes in control cells, myotube formation was significantly decreased upon silencing of Bach1 as judged by morphology, expression of myosin heavy chain (MHC) which are differentiation markers of myoblasts, and a degree of fusion of differentiating cells (Fig 6E–6G). Furthermore, the amount of Bach1 protein was significantly increased in myotubes compared with surrounding undifferentiated cells (Fig 7A).

Consistent with these observations, mRNAs of several MHC family members were decreased upon Bach1 silencing (Fig 7B). Induced expression of MyoD and Myogenin was also reduced upon Bach1 silencing in these cells (Fig 7C). These results indicate that Bach1 plays important roles in myoblast proliferation and differentiation. Since the expression of the myogenic transcription factors was not reduced in the cardiotoxin model of muscle regeneration in *Bach1*-deficient mice, there may be a compensatory regulation at the organismal level.

**Fig 8:**
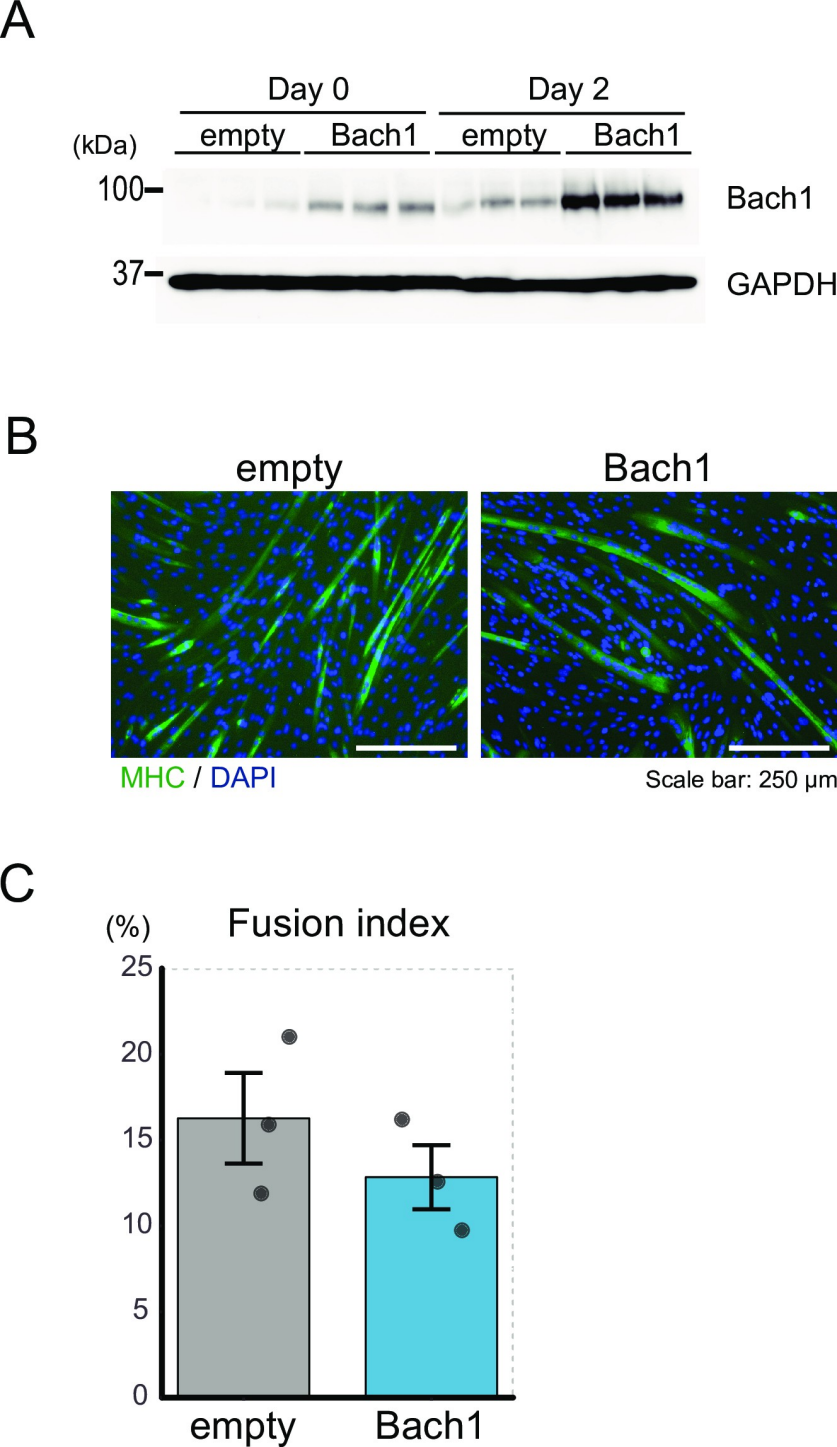
Overexpression of Bach1 does not affect differentiation of C2C12 cells. (A) Western blotting of control and Bach1-overexpressing C2C12 cells before (day 0) and after inducing differentiation (day 2). (B) Immunohistochemical staining of MHC and nuclei (DAPI) of control and Bach1-overexpressing C2C12 cells 5 days after inducing differentiation. (C) Fusion index after 5 days. (n = 3).

We also examined effects of Bach1-overexpression on C2C12 cells (Fig 8A). However, there was no difference of myotube formation between Bach1-overexpressing and control C2C12 cells (Fig 8B and 8C).

### Bach1 does not inhibit senescence in skeletal muscle differentiation

**Fig 9:**
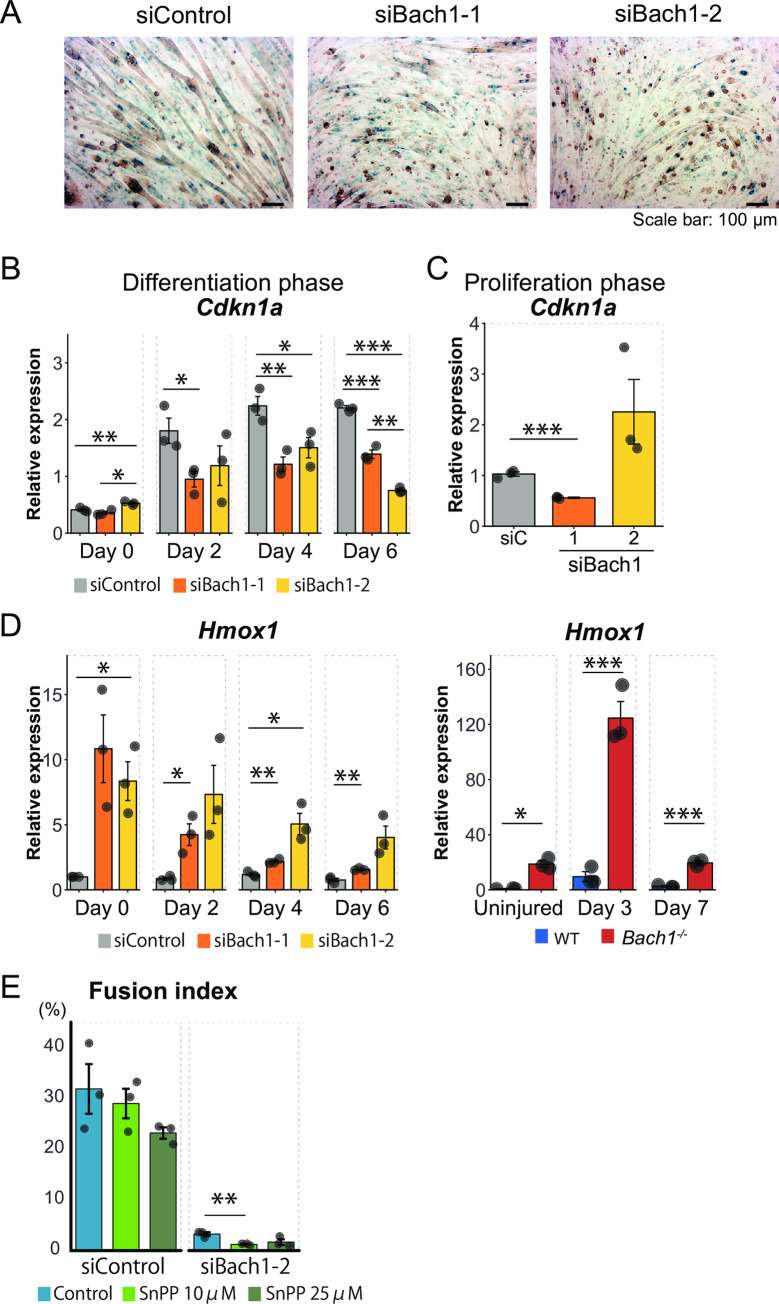
Senescence and HO-1 overexpression of Bach1-silenced cells are not the cause of disturbed differentiation. (A) Bach1-silenced and control C2C12 cells were stained for SA β-gal 6 days after inducing differentiation. (*n* = 3). (B, C)Expression levels of *Cdkn1a* (p21) measured by RT-qPCR at differentiation phase (B) and proliferation phase (C). (*n* = 3).(D) Expression level of *Hmox1* in Bach1-silenced and control C2C12 cells before (day 0) and after inducing differentiation (Day 2, 4 and 6) (left), and of muscle of *Bach1*-deficient and WT before (day 0) and 3 or 7 days after muscle injury (right). (*n* = 3). (E) Fusion index of Bach1-silenced and control C2C12 cells 6 days after inducing differentiation. (*n* = 3; * *p*< 0.05, ** *p*< 0.01, *** *p* < 0.001).

We addressed the possibility that deficiency of Bach1 in C2C12 cells causedenhanced cellular senescence which would affect proliferation and/or differentiation. Enzymatic activity of SA β-gal, a marker of senescence, did not appreciably increase by silencing of Bach1 after inducing differentiation (Fig 9A). Expression of p21 (*Cdkn1a*) [34, 35], one of the targets of p53 and regulators of senescence, was induced upon induction of differentiation, which was rather decreased in the cells with Bach1 silencing (Fig 9B). In contrast, the expression ofp21 in the proliferation phase did not show a consistent response tothe two siBach1 RNAs (Fig 9C). p19 (*Cdkn2a*), another regulator of senescence, was not detected because C2C12 cells lack p19 [36]. We concluded that Bach1 did notinhibit senescence in C2C12 cells, which is in contrast to murine embryonic fibroblasts [21].

### Inhibition of HO-1does not promote skeletal muscle differentiation upon Bach1 silencing

Recently, it has been reported that HO-1 acts asan inhibitor of muscle cell differentiation [37, 38]. Because Bach1 is a direct repressor of HO-1 transcription, we investigated whether Bach1 controlled the expression of HO-1 in muscle cells. The amount of HO-1 mRNA remained almost unchanged in the control C2C12 cells after inducing differentiation (Fig 9D). HO-1 mRNA was strongly increasedupon silencing of Bach1 in C2C12 cells before and after differentiation (Fig 9D, left). Similar results were also obtained in vivo: whereas HO-1 mRNA expression was up-regulated in 3 days after muscle injuryin WT mice, the amount of HO-1 mRNA was substantially much higher in *Bach1*-deficient mice (Fig 9D, right). These results indicate that Bach1 inhibits HO-1 mRNA transcription in both C2C12 cells and skeletal muscle cells. To examine whether the increased expression of HO-1 affected muscle differentiation of myoblast upon Bach1 silencing, we examined the effect of HO-1 inhibitor. After inducing differentiation, C2C12 cells were cultured with tin protoporphyrin IX (SnPP), an inhibitor of HO-1. HO-1 inhibition did not affect muscle differentiation of the control C2C12 cellsand failed to restore muscle differentiation of the cells with Bach1 silencing (Fig 9E). Therefore, we conclude that Bach1 regulates myoblast differentiation byan HO-1 independent pathway.

### Transcriptome alterations in myoblasts upon Bach1 silencing

**Fig 10:**
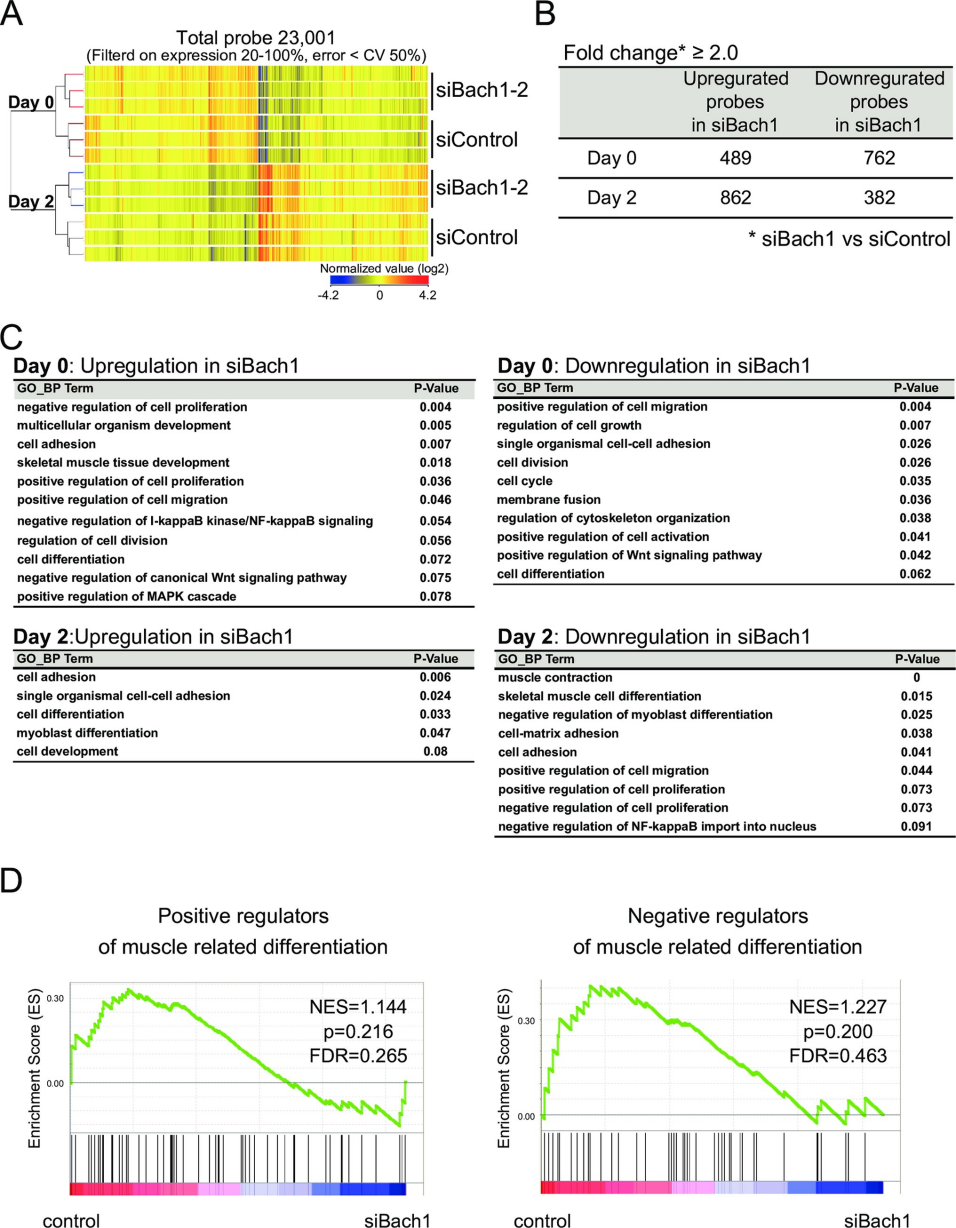
Gene expression profiles of Bach1-silenced and control C2C12 cells. (A) Clustering analysis of microarray gene expression profiles of indicated cells before or after inducing differentiation. (B) The table shows numbers of upregulated and downregulated probes (fold change ≥ 2.0). (C) The tables show gene ontology (GO) analysis results using genes in (B). (D) GSEA enrichment plots of genes related to positive (right) and negative (left) regulators of muscle differentiation.

**Fig 11:**
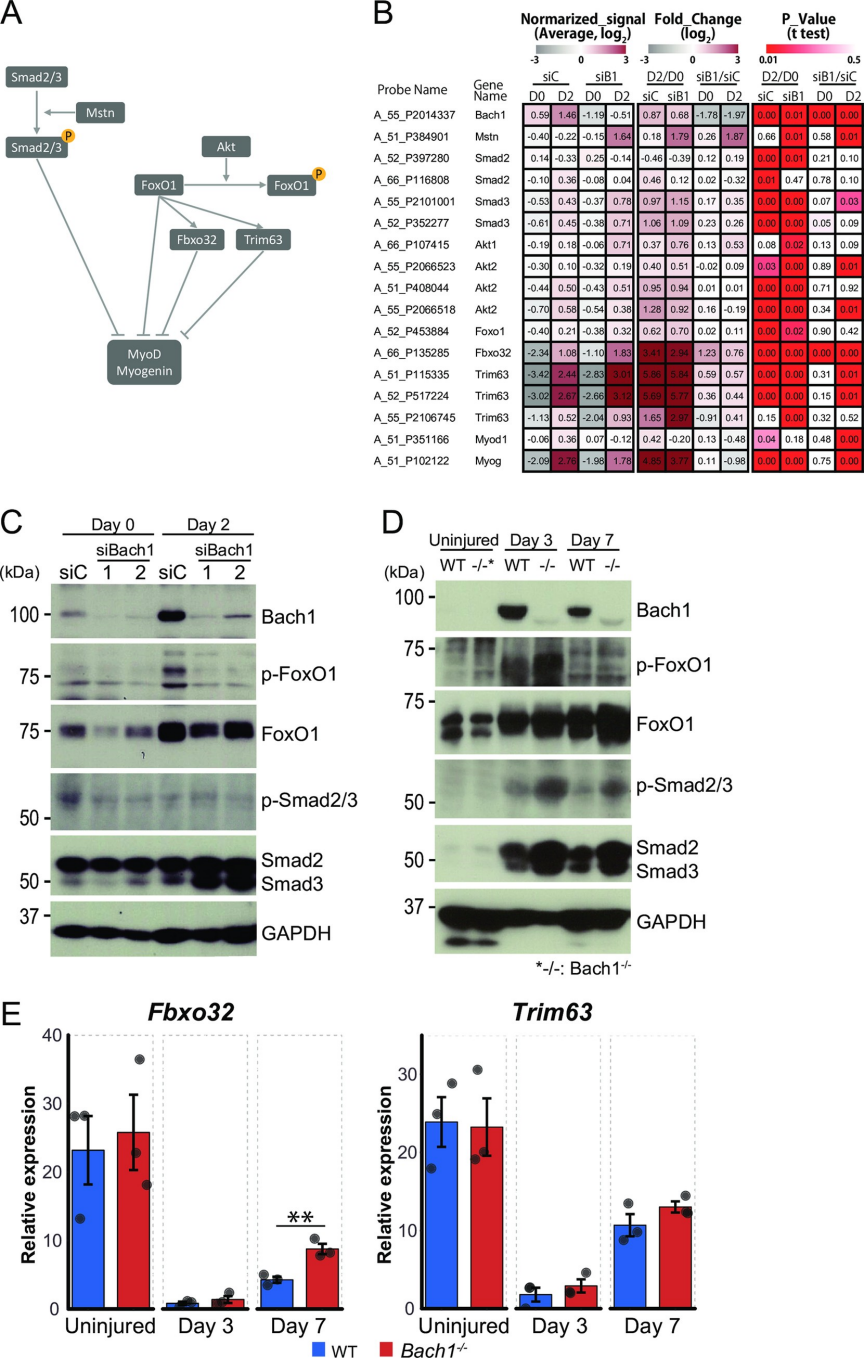
Bach1 decreases Smad3 protein. (A) Signaling pathways known to suppress MyoD and myogenin. (B) Heat map of pathway related genes from microarray results in Fig 10A. Maps show normalized signals (left), fold change (middle) and *p* value (right) compared Bach1-silenced to control C2C12 cells at day 0 and day 2, or compared day 2 to day 0 in Bach1-silenced or control C2C12 cells. (C) Western blotting of Bach1-silenced and control C2C12 cells before (day 0) and after inducing differentiation (day 2). (D) Western blotting of *Bach1-*deficient and WT mice muscle at 3 or 7 days after injury. Uninjured samples are also shown. Western blotting was performed with indicated antibodies. (E) Expression of *Fbxo32* and *Trim63* mRNA measured by RT-qPCR in muscle of *Bach1*-deficient and WT mice at 3 or 7 days after injury or before injury. (*n* = 3; ** *p*< 0.01).

To investigate the Bach1 function in proliferation and differentiation of myogenic cells, we performed DNA microarray analysis using C2C12 cells with silencing of Bach1 (siBach1-2)and control cells before and after inducing differentiation. In a total of 23,001 probes with significant signals (Fig 10A), 489 and 762 genes were up-regulated and down-regulated, respectively, by Bach1 silencing before inducing differentiation (Fig 10B). Upon differentiation, 862 and 382 genes were up-regulated and down-regulated, respectively (Fig 10B). We performed gene ontology (GO) analysis of genes with significant differentialexpression upon Bach1 silencing to investigate its function. Before inducing differentiation, terms related to proliferation were frequently found in both up- and down-regulated genes (Fig 10C). After inducing differentiation, terms related to differentiation were included in both up-regulation and down-regulation groups (Fig 10C). However, GSEA showed no enrichment of genes related to muscle cell differentiation (Fig 10D). Therefore, we examined for possible alterations of known key regulators of muscle differentiation in the microarray data set (Fig 11A).

Smad2, Smad3 and FoxO1 are known to inhibit the expression of MyoD and Myogenin transcription factors [39–41] (Fig 11A). The microarray data analysis revealed that Smad3, one of the key inhibitors of muscle differentiation, wasslightly but reproducibly increased in the cells with Bach1 silencing after inducing differentiation (Fig 11B). Interestingly, Smad3 protein was increased by the combination of Bach1 knockdown and differentiation in C2C12 cells (Fig 11C). Furthermore, not only Smad3 but also Smad2 showed more increase upon cardiotoxin-induced injury in *Bach1*-deficient mice than in WT mice (Fig 11D). In addition, phosphorylated, activated form of Smad3 increased morein the muscle of *Bach1*-deficient mice after injury (Fig 11D). While the amount of FoxO1 protein increased after induction of differentiation of C2C12 cells, its induction was largely not affected by Bach1 silencing in C2C12 cells. In contrast, FoxO1 protein was increased more in the *Bach1*-deficient muscle after the injurythan in the WT muscle (Fig 11D). While the inhibitory phosphorylation of FoxO1 protein was reduced in Bach1 knockdown C2C12 cells, such an effect was not observed in the muscle injury model (Fig 11C and 11D). These alterations were accompanied with changes in the expression of downstream target genes. Expression of *Fbxo32* and *Trim63*, downstream genes of FoxO1, was increasedin C2C12 cells by Bach1 silencing (Fig 11B). Expression of *Fbxo32* was also higher in *Bach1*-deficient mice than WT mice at 7 days after injury whereas that of Trim63 was similar in the two types of mice (Fig 11E).

**Fig 12:**
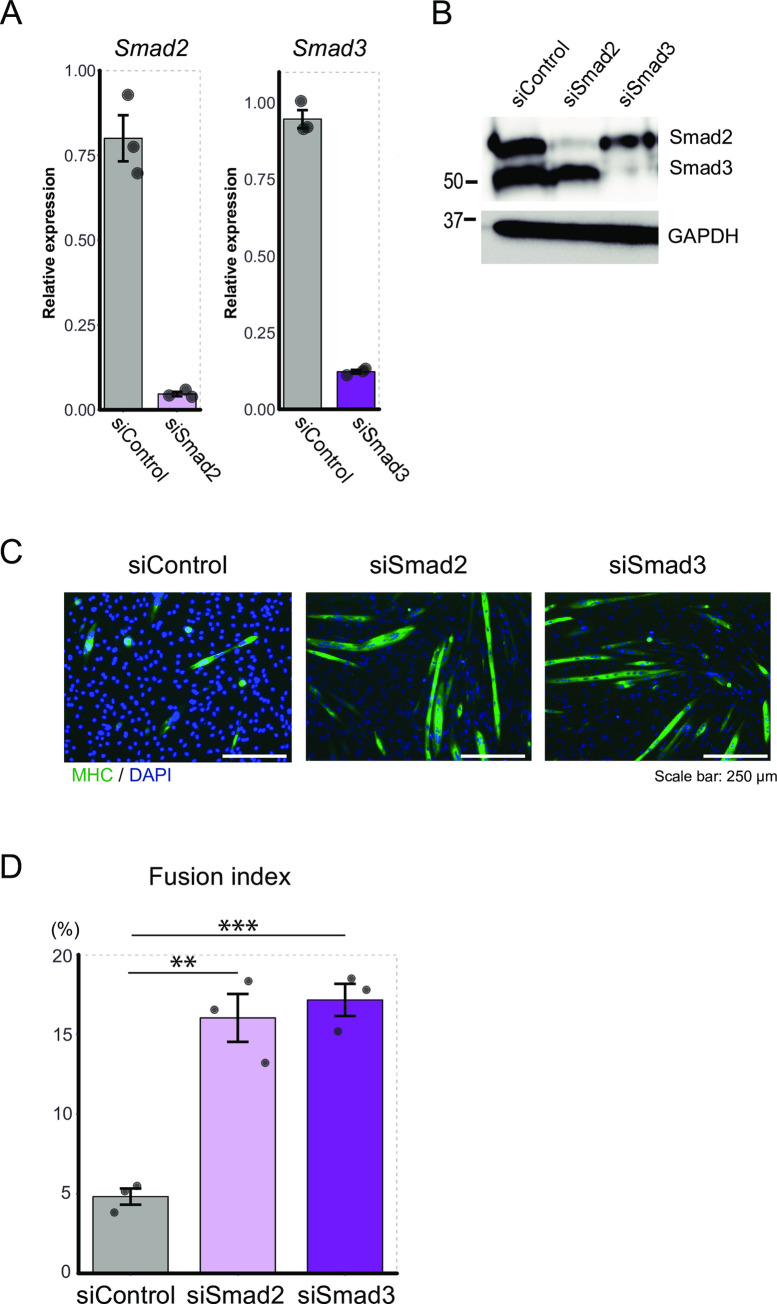
Smad2 and Smad3 inhibit myoblasts differentiation of C2C12 cells. (A) Expression of Smad2 (left) and Smad3 (right) mRNA was measured by RT-qPCR in control C2C12 cells or those treated with indicated siRNA before inducing differentiation. (n = 3). (B) Western blotting of Smad2 and Smad3 in C2C12 cells treated as above after inducing differentiation (day 2). (C) Immunohistochemical staining of MHC and nuclei (DAPI) of C2C12 cells treated as above 5 days after inducing differentiation. (D) Fusion index after 5 days. (n = 3; ** p < 0.01, *** p < 0.001).

**Fig 13:**
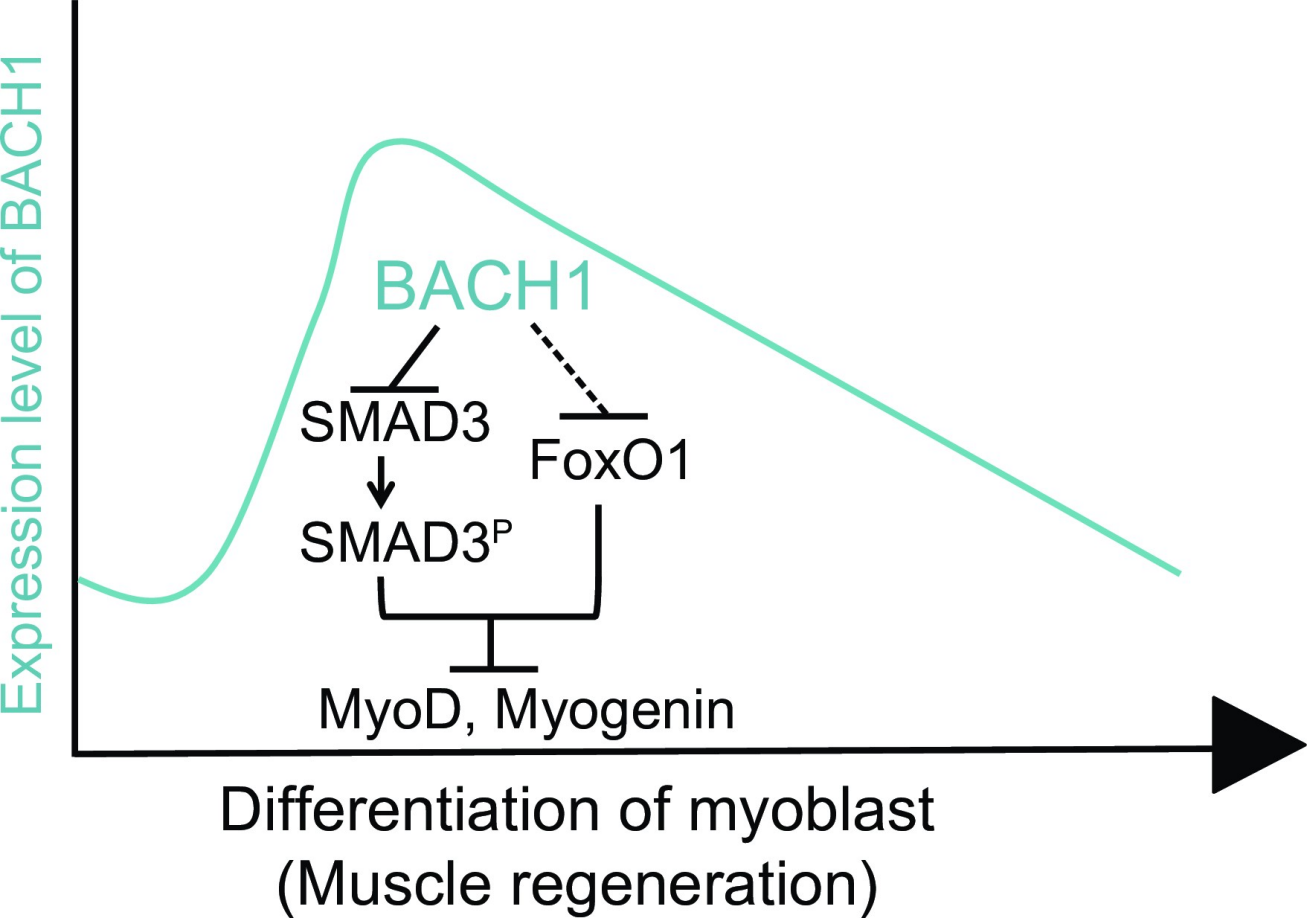
A model on the regulation of myoblast differentiation by Bach1. Bach1 accelerates myoblast differentiation through inhibition of Smad3 and FoxO1 expression.

To validate whether repression of Smad2 or Smad3 by Bach1 was involved in muscle cell differentiation in our experimental system, we performed knockdown of Smad2 or Smad3 in C2C12 cells (Fig 12A and 12B). Myotube formation was significantly increasedbysilencing of Smad2 or Smad3 (Fig 12C and 12D). These results suggested that Bach1 promoted muscle cell differentiation of C2C12 cells and regeneration after muscle injury by directly or indirectly repressing the expressions of Smad2, Smad3 and FoxO1 (Fig 13).

## Discussion

In this report, we found no appreciable differences in muscle weight and muscle fiber size between *Bach1*-deficient and WT mice, indicating that Bach1 is dispensable for the development and maturation of the skeletal muscle system under the normal conditions. Nonetheless, we also found that a critical function of Bach1 in muscle cells manifested after muscle injury, wherethe amount of Bach1 proteinremarkably increased, presumably facilitatingthe required function for efficient muscle regeneration. In terms of molecular mechanism, our results obtained with C2C12 cells and *Bach1*-deficient mice showed altered expression of critical muscle cell regulators that appeared to contribute to the reduced muscle cell differentiation of C2C12 and regeneration in the injury model upon reduction or absence of Bach1 (Fig 11). Amounts of Smad3 protein was increased when Bach1 was reduced. Smad3 might bea direct target of Bach1 becauseour previously data showed Bach2, which is related to Bach1, and MafK, which forms a heterodimer with Bach1, bind to genome region of Smad3 gene body in pre-pro B cell (GSE87503). In addition to Smad3, Smad2 protein was also increased in *Bach1*-deficient, injured muscle. Since Bach1 directly represses the expression of *Nodal* gene which encodes one of the ligands for the Smad pathway in human embryonic stem cells [42], Bach1 may regulate Smad pathway at multiple steps. Consistent with the previous report that Smad2 and Smad3 inhibit MyoD and myogenin expression [39], the expression of the two myogenic transcription factors was reduced in C2C12 cells upon knockdown of Bach1. However, the expression of these myogenic transcription factors was not affected in *Bach1*-deficient mice. Therefore, Smad2 and Smad3 may inhibit the expression of additional target genes other than these myogenic transcription factors. In addition, increased activity of FoxO1, which was suggested by the reduction of phosphorylated FoxO1, may be relevant to the differentiation defect of C2C12 cells with Bach1 silencing, since FoxO1 is known to inhibit the expression of MyoD and myogenin [40, 43]. Even though such reduction of phosphorylated FoxO1 was not observed in the muscle of *Bach1*-deficient mice, total amount of FoxO1 protein increased more in *Bach1*-deficient mice than wild-type mice at 7 days after injury. FoxO1 is known to regulate muscle atrophy by inducing Fbxo32 [44], which was also increased more in *Bach1*-deficient muscle compared with the control tissue. Bach1 may regulate not only differentiation of myoblasts but also hypertrophy of myofibers by affecting FoxO1.

Amounts of both Bach1 mRNA and protein were increasedshortly after induction of differentiation in C2C12 cells whereas only the protein was reduced following its initial induction. Considering the effect of Bach1 upon the expression of Smad2 and Smad3, the returning of Bach1 protein to its basal level may restrict myotube formation. However, a continuous Bach1 overexpression did not increase myotube formation. One possibility is that overexpressed Bach1 was degraded by a post-translational regulation which was induced at the later stage of differentiation. We observed that Bach1 protein was increased in muscle injury without clear increase of Bach1 mRNA. These observations suggest that post-translational regulation of Bach1 is operating in injured muscle cells. It will be important to unravel the presumptive degradation system of Bach1 toward better understanding the regulation of myotube formation and regeneration.

Our findings on the muscle injury model are in clear contrast to previous reports showing reduction of myocardial infarction, spinal injury, and lung injury by the *Bach1* deficiency in disease models [9–11]. One of the possible reasons may be the unique feature of skeletal muscle regeneration. In skeletal muscle, damaged muscle fibers are removed and satellite cells, but not muscle fiber itself, regenerate new muscle fibers after injury-induced expansion and differentiation into myoblasts. Oxidative damage may rather work favorably to decompose damaged muscle fibers and to accelerate repair processes of the muscle. Indeed, many inflammatory cells migrated to the injured muscle within 3 days after the injury, and most of muscle fibers were replaced by reproduced fibers that have central nuclei. To address this possibility further, it will be important to examine whether inhibition of HO-1 in *Bach1*-deficient mice promotes regeneration. Characteristics of the injury model employed here may also contribute to the unique function of Bach1 in muscle regeneration. There are several models for skeletal muscle injury like freezing [45], contusion [46], muscle strain [47], and muscle cutting [48]. Chemical damage induced by cardiotoxin is a model with relatively small damage for satellite cells [45, 49]. In *Nrf2*-deficient mice, muscle regeneration is impaired after ischemia-reperfusion injury [50]. Since Nrf2 activates genes many of which are repressed by Bach1 including HO-1, *Bach1-*deficient mice may show an increase in regeneration in such a model. In models of ischemia-reperfusion injury and muscular dystrophy, HO-1 deficiency causes a decrease of regeneration capacity [51, 52]. Since these models cause strong oxidative stress [53, 54], muscle regeneration may be impaired by damagingnot only muscle cells but also satellite cells. Thus, the regeneration-promoting function of Bach1 in muscle may manifest on the balance between enhanced anti-oxidative stress response and decreased myoblast activity.

Overexpression of HO-1 in C2C12 cells has been reported to inhibit differentiation [37]. While we found that Bach1 knockdown in C2C12 cells led to higher expression of HO-1, inhibition of HO-1 in these cells did not result in any recovery of differentiation. This is consistent with our findings which suggest the presence of additional target genes of Bach1 like Smad proteins in muscle cells. Therefore, our observations do not necessarily negate the inhibitory role HO-1in muscle differentiation of C2C12 cells.

Sarcopenia, an age-related degenerative loss of skeletal muscle mass and strength, is one of the major factors of functional decline of elderly people [55]. Sarcopenia is correlated with muscle injury and impaired muscle regeneration [55, 56]. Therefore, our results suggest that Bach1 may also be involved in sarcopenia. However, there are several limitations to this present study in terms of muscle cell physiology at an organismal level. Rodents models are constrained by their marked differences in metabolic and endocrine pathways with humans [57]. While the observations presented here strongly support a function of Bach1 in muscle cell differentiation or homeostasis, they do not exclude its possible function in non-muscle cells that may facilitate muscle cell differentiation in a non-cell autonomous manner. Further studies using new animal models more relevant to human physiology, such as a swine model [58], and tissue-specific knockout of Bach1 will be necessary to address these issues. Nonetheless of these limitations, our observations using C2C12 cells suggest that Bach1 promotes muscle cell differentiation by repressing directly or indirectly downstream regulators such as Smad2 and Smad3, tuning the process of muscle differentiation.

## Materials and methods

### Mice

All the animal experiments were approved by the Institutional Animal Care and Use Committee of the Tohoku University Environmental and Safety Committee (No. 2016MdA-213). We used *Bach1*-deficient and WT mice with C57B6J background [59]. Genotyping was performed using DNA extraction from tail of mice. PCR for genotyping were carried out by using following primers.

*Bach1* wild-type (forward) 5’-CATGTGTGTTTGCAGGTCGA-3’

wild-type (reverse) 5’-GTGGAAGTAGCTGCTGCACG-3’

mutant (forward) 5’-CATGTGTGTTTGCAGGTCGA-3’

mutant (reverse) 5’-AGTAGGTGTCATTCTATTCTGGG-3’

### Animal procedures

For cardiotoxin induced muscle injury, mice were anesthetized with an intraperitoneal injection of 200 μl of normal saline containing medetomidine hydrochloride (0.6 mg/ml) and midazolam (8 mg/ml), and 50 μl of buprenorphine(0.05 mg/ml). After removing their hind legs hair with depilatory, 50 μl cardiotoxin (10 μM) from Naja pallida (Latoxan L8102) were administered intramuscularly into the right tibialis anterior (TA) muscle in five positions to assure distribution of cardiotoxin all over the muscle. Mice were sacrificed at day 3, 7, 21 after cardiotoxin treatment and before treatment. The bilateral TA were collected, weighted and frozen immediately in isopentane cooled in liquid nitrogen. Frozen TA muscles were stored at -80°C until use. These were snap frozen in OCT compound (Sakura Fine technical) and sectioned at a thickness of 10 μm with a cryostat (Leica).

### Histology and immunohistochemistry

Muscle sections were stained with hematoxylin and eosin (HE) or immunostained with antibodies. For immunostaining, sections were air dried and washed in PBS, blocked for 1 hr with 1% BSA/PBS, and were incubated with rabbit anti-Laminin polyclonal antibody (1:1000 dilution, ab11575, Abcam) or rabbit anti-Bach1 polyclonal antibody (1:400 dilution) [18] for 30 min. Sections were then washed with PBS and incubated with secondary antibody conjugated with Alexa Fluor 488 (1:1000 dilution, A-21206, Thermo Fisher Scientific) or FITC (1:1000 dilution, 65–6111, Invitrogen) for 30min. After washing, sections were mounted in VECTASHIELD Mounting Medium with DAPI (VECTOR) or Hoechst.

### Cross sectional area measurement

CSA in each fiber was measured in 5 fields of vision of muscle sections that were stained with laminin antibody in each mouse. Each section was automatically outlined and measured areas of each circle using Photoshop CC (Adobe). In injured mice, only circles with central nucleiwere measured.

### Cell culture

C2C12 myoblast cells were purchased from Riken Cell Bank (Japan). Cells were cultured at 37°C in 5% CO_2_ in high-glucose Dulbecco’s modified Eagle medium (DMEM, Sigma) containing 10% fetal bovine serum and 1% penicillin/streptomycin (10,000 units of penicillin per ml, 10,000 μg of streptomycin per ml) (GIBCO). C2C12 cells were passaged before confluence. C2C12 cells were induced to differentiate into myotubes by replacing in the DMEM with 2% horse serum (HS) when the cells reached confluence. For proteasome inhibition, C2C12 cells were incubated withMG132-containing growth medium for 3 hrs. For HO-1 inhibition treatments, cells were incubated with the HS-containing medium with or without tin protoporphyrin IX (SnPP) for 6 days.

M1 cells were cultured at 37°C in 5% CO_2_ inRPMI 1640 (Sigma) containing 10% fetal bovine serum and 1% penicillin/streptomycin.

### RNA interference

Bach1 was silenced using stealth RNAi^TM^ (Invitrogen). These sequences are as follow.

siBach1-1: UUUCCAAGUUGCUUGAGCAGCCUUC

siBach1-2: UUGAAUGGCAGCUUCACCUCACAGU

siSmad2: UCGGAACCUGCAUUCUGGUGUUCAA

siSmad3: CCUGCUGGAUUGAGCUACACCUGAA

Transfection of small interfering RNA was performed using Lipofectamine RNAiMAX reagent (Invitrogen). Control siRNA (Stealth RNAi^TM^ negative control) was used for comparison. Stealth RNAi was used twice the amount of volume written in protocol. In Smad knockdown experiment, four-fold more amounts of siControl, siSmad2, or siSmad3 were used. The knockdown efficiency was confirmed by Western blotting or qPCR.

### Bach1 overexpression

The Bach1 and FLAG expression plasmids (pCMV-Bach1 and pcDNA3.1B-FLAG, respectively) used were as previously described [60, 61]. C2C12 cells were transfected using GeneJuice (Novagen) according to the reagent protocol. Efficiency was confirmed by Western blotting.

### Immunostaining and quantification of fusion index

C2C12 cells were fixed with 4% paraformaldehyde solution for 10 minutes and permeabilized with 0.5% TritonX-100 (Nakarai Chemicals) and 0.1% SDS solution for 10 min. Cells were incubated with rabbit anti-Bach1 polyclonal antibody (1:400 dilution) [18] or anti-myosin heavy chain antibody (MY32, Sigma) diluted with PBS containing 1% BSA for 1 hr at 37°C. Cells were then incubated with secondary antibody conjugated with sheep anti-mouse IgG-FITC (F3008, Sigma) diluted with PBS containing 1% BSA for 30 min at 37°C. Cells were mounted in VECTASHIELD Mounting Medium with DAPI (VECTOR) or Hoechst. Fusion index was calculated as percentage of nuclei within myosin positive cells with at least 3 nuclei versus total nuclei in the fields.

### Western blotting

Total cell lysates were loaded onto 7.5% agarose gel. Electrophoresis was run and wet transferred to PVDF membrane (Millipore) at 300 mA for 2 hr. After blocking with 5% skim milk powder (Wako) in T-TBS for 1 hr, membranes were incubated with primary antibody solutions at 4°C overnight. Membranes were then washed with T-TBS, and incubated with horseradish peroxidase conjugated anti-IgG antibodies (GE Healthcare) at RT for 1 hr. Antibody binding was detected with a chemiluminescent substrate (Pierce ECL Plus Western Blotting Substrate, Thermo Fisher Scientific) and X-ray film (GE Healthcare). Antibodies used were:rabbit anti-Bach1 polyclonal antibody (1:1000dil) [18], rabbit anti-FoxO1 monoclonal antibody (1:1000 dil, 2880, Cell Signaling), rabbit anti-phospho-FoxO1 polyclonal antibody (1:1000 dil, 9461, Cell Signaling), rabbit anti-Smad2/3 monoclonal antibody (1:300 dil, 8685, Cell Signaling), rabbit anti-phospho-Smad2/3 monoclonal antibody (1:200 dil, 8828, Cell Signaling) and mouse anti-GAPDH monoclonal antibody (1:5000 dil, ab8245, Abcam).

### Quantitative real-time PCR

Total RNA was extracted from C212 cells and skeletal muscle using Rneasy Mini Kit (Qiagen) or Rneasy Micro Kit (Qiagen) according to the protocol. Complementary DNA was synthesized from total RNA using Omniscript Reverse Transcription Kit (Qiagen) or SuperScript III Reverse Transcriptase (Thermo Fisher Scientific) with Random primer (Invitrogen) and RNaseOUT (Thermo Fisher Scientific). qPCR was performed in Light Cycler Nano (Roche) or Light Cycler 96 (Roche) using FastStart Essential DNA Green Master (Roche). Relative expression was calculated using Csnk2a2 cDNA as a control. Primers used are as follows.

Bach1

Forward: 5’-GCCCGTATGCTTGTGTGATT-3’

Reverse: 5’-CGTGAGAGCGAAATTATCCG-3’

*Cdkn1a* (p21)

Forward: 5’-GCAGATCCACAGCGATATCCA-3’

Reverse: 5’-AGACAACGGCACACTTTGCT-3’

*Cdkn2a*(p19)

Forward: 5’-GCTCTGGCTTTCGTGAACA-3’

Reverse: 5’-TCGAATCTGCACCGTAGTTG-3’

Csnk2a2

Forward: 5’-CCACATAGACCTAGATCCACACT-3’

Reverse: 5’-CGCAGGAGCTTGTCAAGAAGA-3’

*Fbxo32*(Atrogin-1)

Forward: 5’-CAGCTTCGTGAGCGACCTC-3’

Reverse: 5’-GGCAGTCGAGAAGTCCAGTC-3’

*Hmox1*(HO-1)

Forward: 5’-GGGTGACAGAAGAGGCTAAG-3’

Reverse: 5’-GTGTCTGGGATGAGCTAGTG-3’

Myf5

Forward: 5’-CACCACCAACCCTAACCAGAG-3’

Reverse: 5’-AGGCTGTAATAGTTCTCCACCTG-3’

*Myf6* (MRF4)

Forward: 5’-ATTCTTGAGGGTGCGGATTTC-3’

Reverse: 5’-CCTTAGCAGTTATCACGAGGC-3’

*Myh1* (MHCⅡd/x)

Forward: 5’-AATCAAAGGTCAAGGCCTACAA-3’

Reverse: 5’-GAATTTGGCCAGGTTGACAT-3’

*Myh7* (MHCⅠ/b)

Forward: 5’-CGCATCAAGGAGCTCACC-3’

Reverse: 5’-CTGCAGCCGCAGTAGGTT-3’

*Myod1* (MyoD)

Forward: 5’-GCAGAATGGCTACGACACC-3’

Reverse: 5’-CCTGTTCTGTGTCGCTTAGG-3’

*Myog* (Myogenin)

Forward: 5’-CCTTGCTCAGCTCCCTCA-3’

Reverse: 5’-TGGGAGTTGCATTCACTGG-3’

Smad2

Forward: 5’-CAACCAGGGTTTTGAAGCCG-3’

Reverse: 5’-TGCAGAGGGCCATTCAGATG-3’

Smad3

Forward: 5’-AAGCTCAAGAAGACGGGGC-3’

Reverse: 5’-CGGCAGTAGATAACGTGAGGG-3’

*Trim63* (MuRF-1)

Forward: 5’-GTGTGAGGTGCCTACTTGCTC-3’

Reverse: 5’-GCTCAGTCTTCTGTCCTTGGA-3’

### Senescence-associated β-galactosidase staining

C2C12 cells were induced to differentiate for 6 days. Senescence-associated β-galactosidase (SA-β gal) staining was performed using Senescence Detection Kit (K320, BioVision) according to the protocol.

### Microarray analysis

The microarray analyses were performed with RNA isolated from C2C12 cells, transfected with siControl and siBach1-2, without or with induction of differentiation (day 0 or day 2). The experiments were carried out in triplicate. Total RNA was extracted using Rneasy Mini Kit (Qiagen). cRNA labeled with Cyanine 3 was synthesized using Low Input Quick Amp Labeling Kit (Agilent). cRNA was hybridized on Sure Print G3 Mouse GE microarrays 8×60K Ver. 2.0 (Agilent). Microarrays were scanned with Agilent DNA microarray scanner (Agilent). Results were analyzed using GeneSpring Ver 14.5 (Agilent), DAVID (https://david.ncifcrf.gov) and gene set enrichment analysis ver. 3.0. Gene set of “Positive regulators of muscle related differentiation” is a combined gene set of GO_POSITIVE_REGULATION_OF_MUSCLE_CELL_DIFFERENTIATION, GO_POSITIVE_REGULATION_OF_MYOBLAST_DIFFERENTIATION, GO_POSITIVE_REGULATION_OF_MYOTUBE_DIFFERENTIATION, and GO_POSITIVE_REGULATION_OF_STRIATED_MUSCLE_CELL_DIFFERENTIATION, and gene set of “Negative regulators of muscle related differentiation” was a combined gene set of GO_NEGATIVE_REGULATION_OF_MUSCLE_CELL_DIFFERENTIATION, GO_NEGATIVE_REGULATION_OF_MYOBLAST_DIFFERENTIATION, GO_NEGATIVE_REGULATION_OF_MYOTUBE_DIFFERENTIATION, and GO_NEGATIVE_REGULATION_OF_STRIATED_MUSCLE_CELL_DIFFERENTIATION.

### Statistics

Results were averaged and utilized to identify statistical significance using Student’s t tests. Statistical significance was set as *p*< 0.05.

## Supporting information

S1 Raw Images (PDF)

## Abbreviations

Bach1: BTB and CNC homology 1
CSA: Cross sectional area
Cdkn1a/2a: Cyclin dependent kinase inhibitor 1a / 2a
Fbxo32: F-box protein 32
FoxO1: Forkhead box O1
GAPDH: Glyceraldehyde-3-phosphate dehydrogenase
GO: Gene ontology
GSEA: Gene set enrichment analysis
HDAC1: Histone deacetylase 1
HO-1/Hmox1: heme oxygenase-1
HS: Horse serum
Maf: Musculoaponeurotic fibrosarcoma
MHC/Myh: myosin heavy chain
MyoD/Myod1: Myogenic differentiation 1
Myf5/6: Myogenic factor 5/6
MARE: Maf recognition element
MRF: Myogenic regulatory factor
MRF4: Muscle-specific regulatory factor 4
MuRF-1: Muscle-specific RING finger protein 1
Nrf2: Nuclear factor erythroid 2-related factor 2
SA β-gal: Senescence-associated beta-galactosidase
Smad2/3: SMAD family member 2 / 3
TA: Tibialis anterior
Trim63: Tripartite motif containing 63
