# Supplementary figures and images for: Bach1 promotes muscle regeneration through repressing Smad-mediated inhibition of myoblast differentiation
Source: PLoS One. 2020 Aug 10;15(8):e0236781. doi: 10.1371/journal.pone.0236781 (PMC7416950; doi:10.1371/journal.pone.0236781)

Fig 3

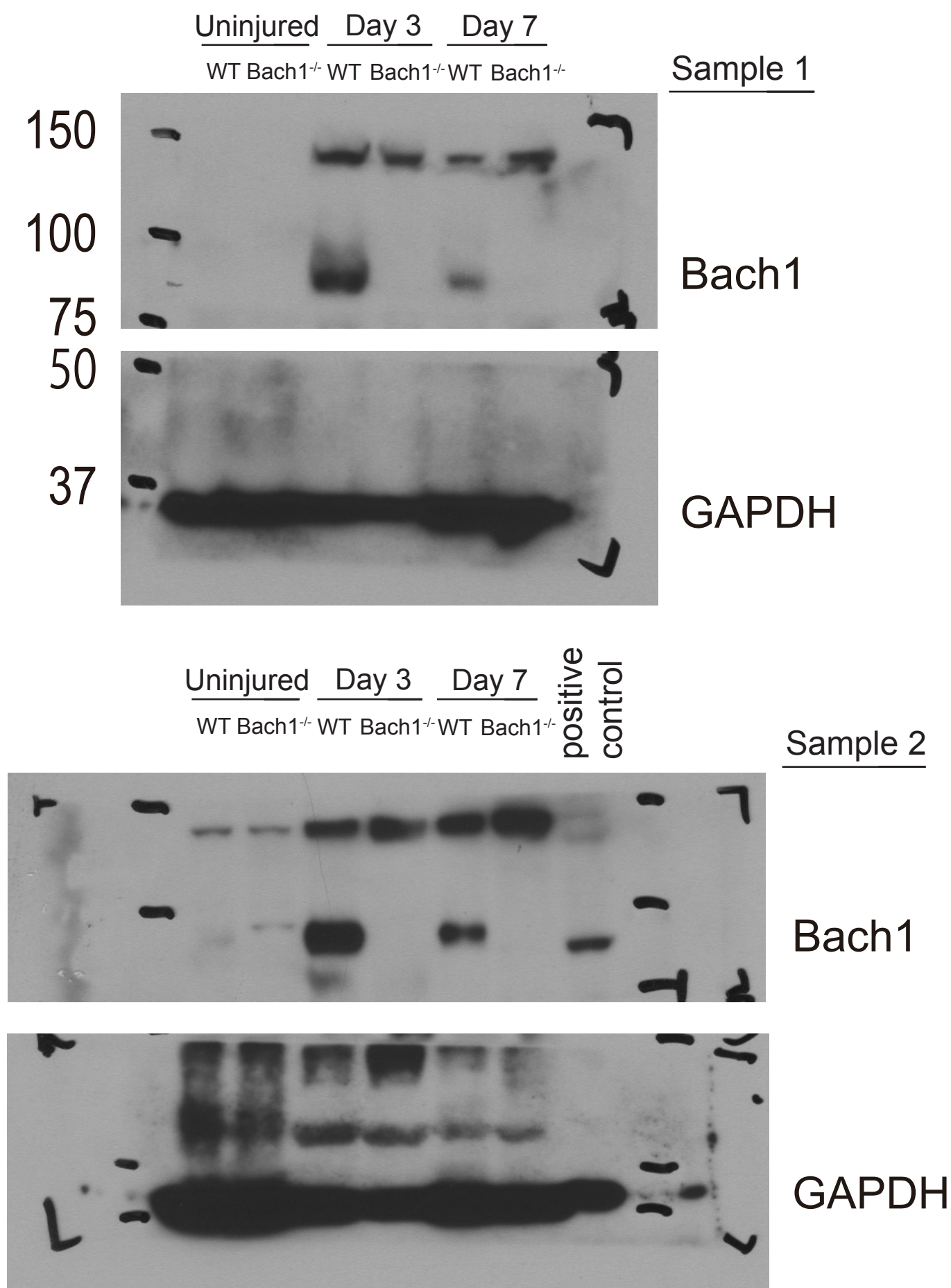

Fig 4

Marker                M1 cells                          C2C12 cells                Marker

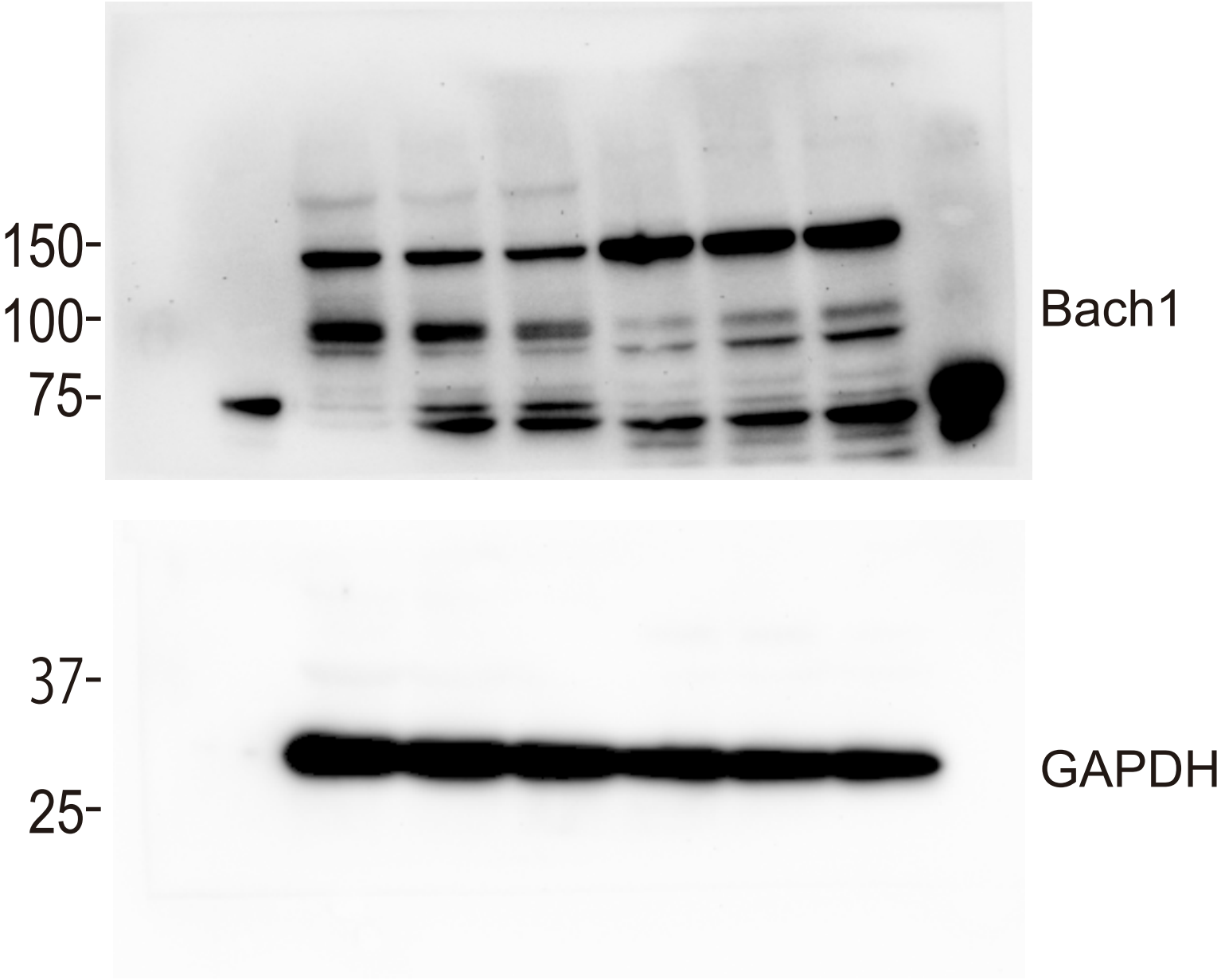

Fig 6

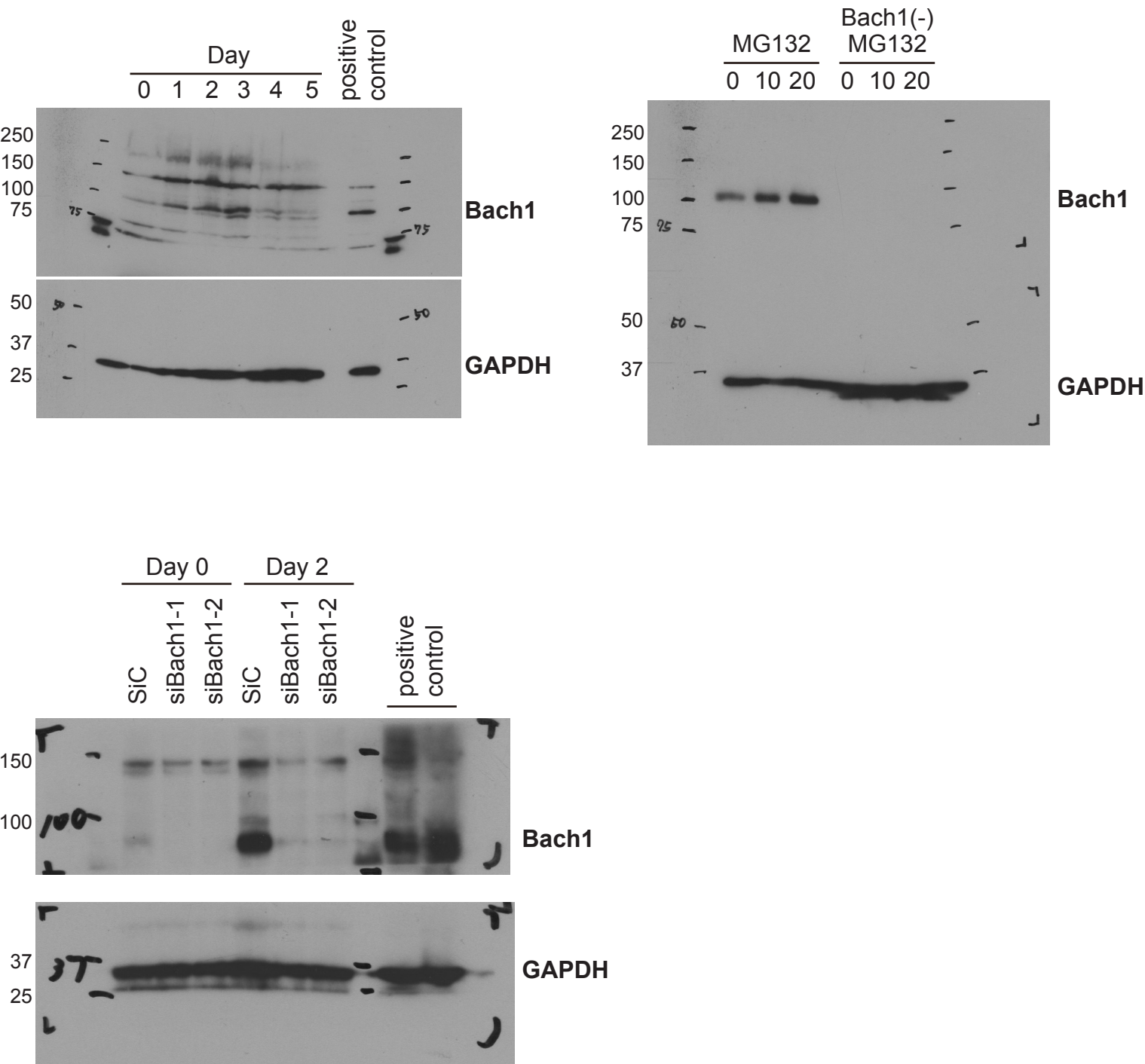

Fig 8

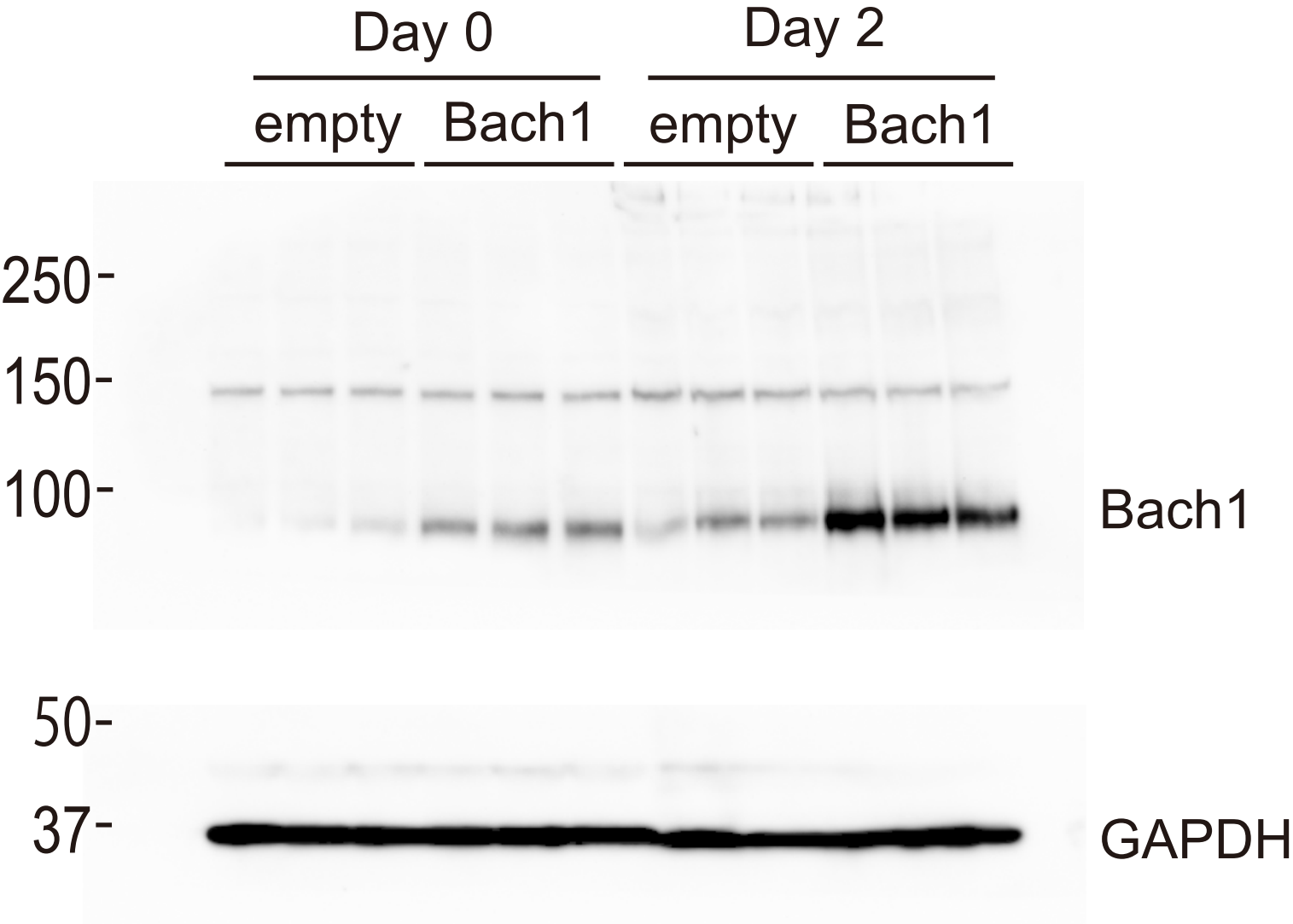

Fig 11

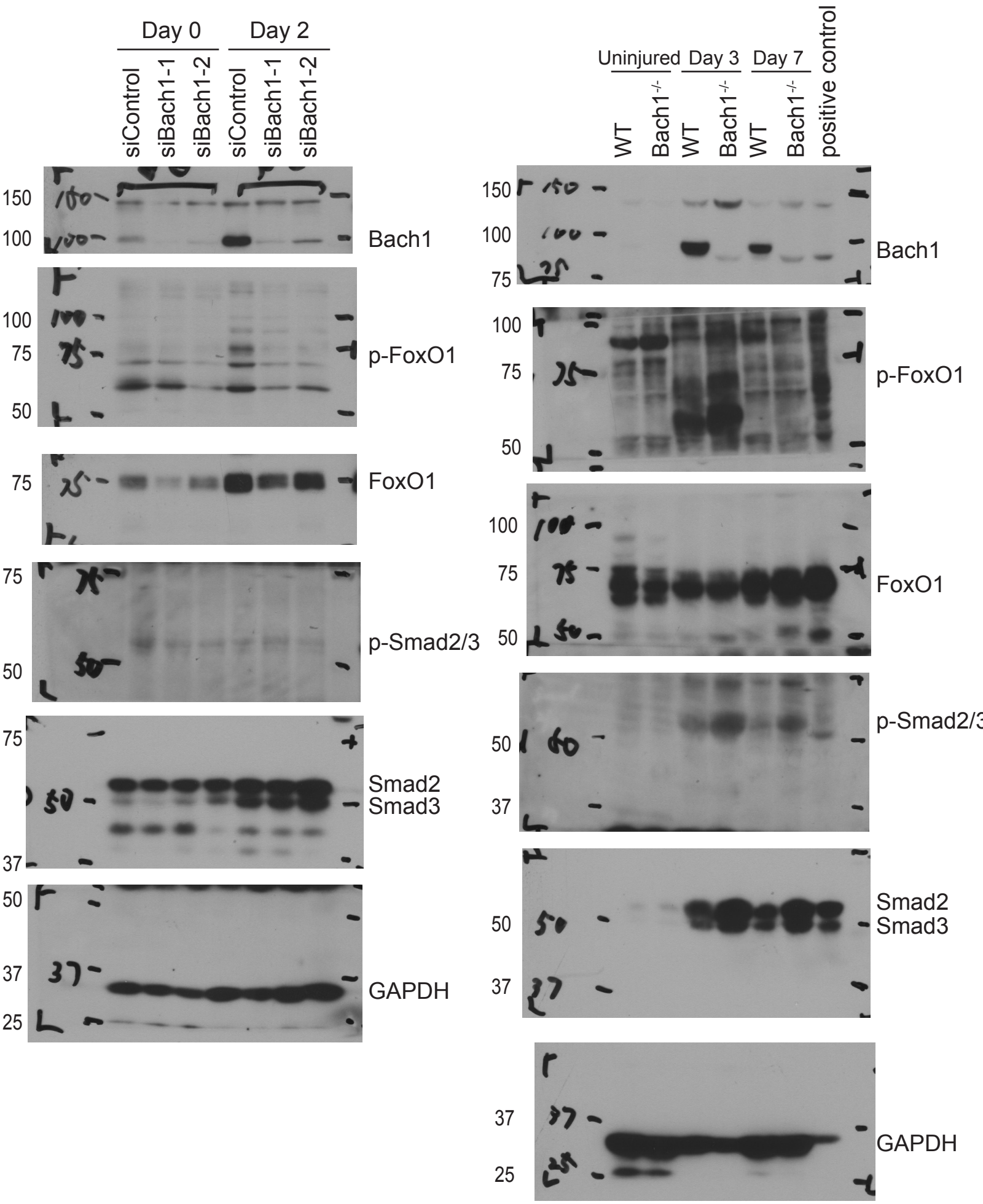

Fig 12

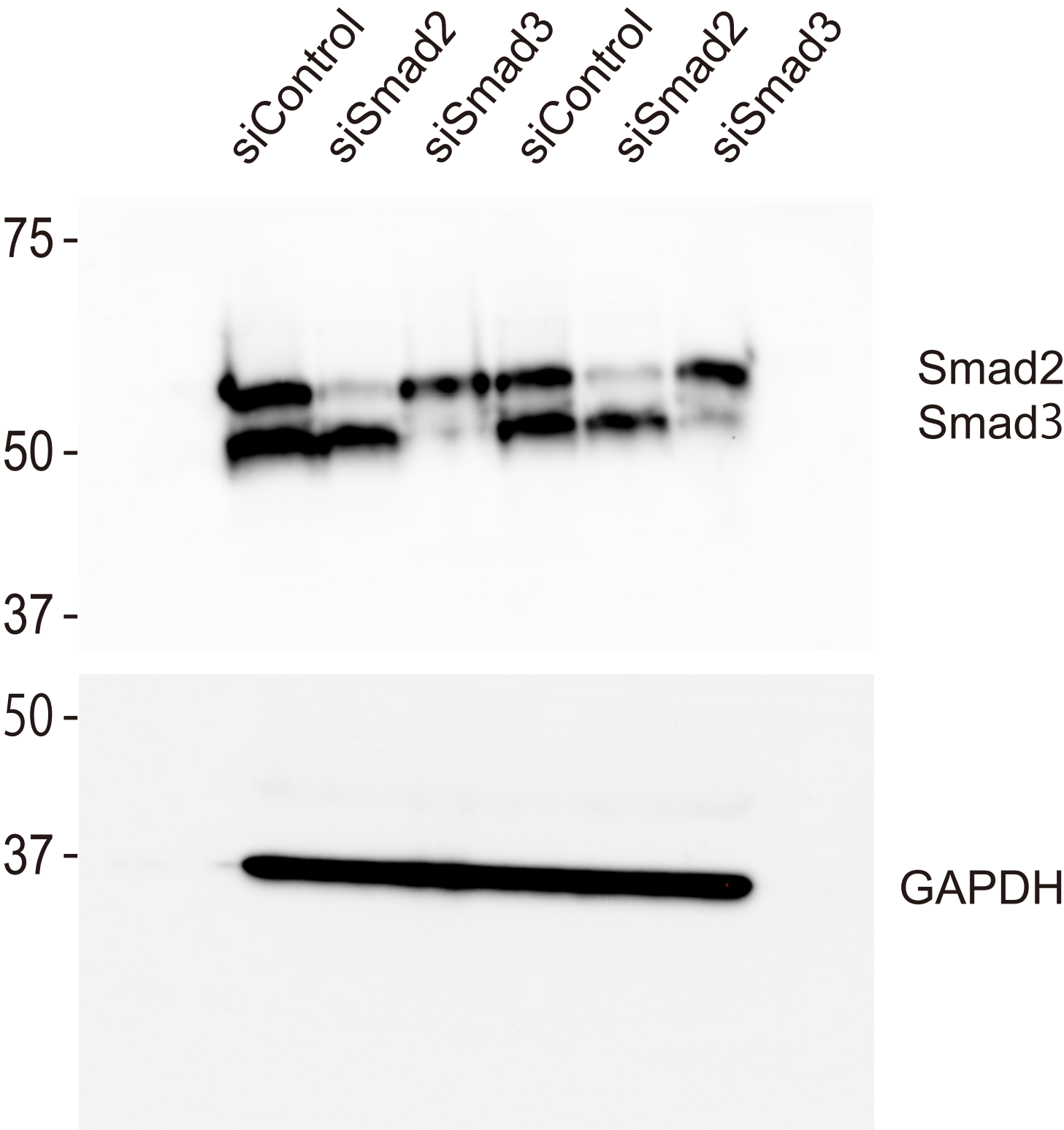

Supplement: S1 Raw Images — (PDF) [file pone.0236781.s001.pdf]
